# Breast Cancer during Pregnancy as a Special Type of Early-Onset Breast Cancer: Analysis of the Tumor Immune Microenvironment and Risk Profiles

**DOI:** 10.3390/cells11152286

**Published:** 2022-07-24

**Authors:** Elham Sajjadi, Konstantinos Venetis, Marianna Noale, Hatem A. Azim, Concetta Blundo, Giuseppina Bonizzi, Eugenia Di Loreto, Giovanna Scarfone, Stefano Ferrero, Stefania Maggi, Massimo Barberis, Paolo Veronesi, Viviana E. Galimberti, Giuseppe Viale, Nicola Fusco, Fedro A. Peccatori, Elena Guerini-Rocco

**Affiliations:** 1 Department of Oncology and Hemato-Oncology, University of Milan, Via Festa del Perdono 7, 20122 Milan, Italy; elham.sajjadi@unimi.it (E.S.); konstantinos.venetis@unimi.it (K.V.); paolo.veronesi@unimi.it (P.V.); giuseppe.viale@unimi.it (G.V.); elena.guerini@unimi.it (E.G.-R.); 2 Division of Pathology, IEO, European Institute of Oncology IRCCS, Via Giuseppe Ripamonti 435, 20141 Milan, Italy; giuseppina.bonizzi@ieo.it (G.B.); massimo.barberis@unimi.it (M.B.); 3 National Research Council (CNR), Neuroscience Institute Aging Branch, Via Giustiniani 2, 35128 Padua, Italy; marianna.noale@in.cnr.it (M.N.); stefania.maggi@in.cnr.it (S.M.); 4 Breast Cancer Center, Hospital Zambrano Hellion, Tecnologico de Monterrey, San Pedro Garza Garcia 66278, NL, Mexico; hatem.azim@gmail.com; 5 Breast Surgery Unit, Fondazione IRCCS Ca’ Granda—Ospedale Maggiore Policlinico, 20122 Milan, Italy; concetta.blundo@policlinico.mi.it; 6 Gynecology Unit, Fondazione IRCCS Ca’ Granda—Ospedale Maggiore Policlinico, 20122 Milan, Italy; diloreto.eugenia@gmail.com (E.D.L.); giovanna.scarfone@policlinico.mi.it (G.S.); 7 Division of Pathology, Fondazione IRCCS Ca’ Granda—Ospedale Maggiore Policlinico, 20122 Milan, Italy; stefano.ferrero@policlinico.mi.it; 8 Department of Biomedical, Surgical, and Dental Sciences, University of Milan, Via Festa del Perdono 7, 20122 Milan, Italy; 9 Division of Breast Surgery, IEO, European Institute of Oncology IRCCS, 20141 Milan, Italy; viviana.galimberti@ieo.it; 10 Fertility and Procreation Unit, Division of Gynecologic Oncology, IEO European Institute of Oncology IRCCS, 20141 Milan, Italy; fedro.peccatori@ieo.it

**Keywords:** breast cancer during pregnancy, early-onset breast cancer, pregnancy-related breast cancer, tumor-infiltrating lymphocytes, PD-L1, tumor microenvironment, breast cancer, biomarkers

## Abstract

Breast cancer during pregnancy (PrBC) is a rare tumor with only a little information on its immune landscape. Here, we sought to characterize the cellular composition of the tumor microenvironment (TME) of PrBC and identify its differences from early-onset breast cancer (EOBC) in non-pregnant women. A total of 83 PrBC and 89 EOBC were selected from our Institutional registry and subjected to tumor-infiltrating lymphocytes (TILs) profiling and immunohistochemistry for CD4, CD8, forkhead box P3 (FOXP3), and programmed death-ligand 1 (PD-L1) (clone 22C3). A significantly lower frequency of hormone receptor (HR)-positive tumors was observed in PrBC. The prevalence of low/null PD-L1 and CD8+TILs was higher in PrBC than in the controls, specifically in HR+/HER2– breast cancers. PrBC had a significantly higher risk of relapse and disease-related death, compared to EOBC. The presence of TILs and each TIL subpopulation were significantly associated with disease relapse. Moreover, the death rate was higher in PrBC with CD8+ TILs. The TME of PrBC is characterized by specific patterns of TIL subpopulations with significant biological and prognostic roles. Routine assessment of TILs and TILs subtyping in these patients would be a valid addition to the pathology report that might help identify clinically relevant subsets of women with PrBC.

## 1. Introduction

Breast cancer is one of the most common malignancies occurring during pregnancy, with approximately 1400 new diagnoses every year in Europe [1,2]. This condition, commonly referred to as breast cancer during pregnancy (PrBC) accounts for ~4% of early-onset breast cancers (EOBC), i.e., breast cancer diagnosed in pre-menopausal women aged 18–45 years [3]. Despite being relatively rare in the general population (140 per 100,000 pregnancies), PrBC prevalence is steadily rising [4,5]. Overall, PrBC is associated with a relatively more aggressive clinical behavior compared to breast cancer and EOBC [6,7,8,9,10]. It is recommended to follow standard treatment guidelines for these patients, but it is worth mentioning that the feasibility of novel/emerging treatment protocols (e.g., immunotherapy, targeted therapy, antibody-drug conjugates) has yet to be assessed in PrBC because of the lack of dedicated clinical trials [11].

From a clinicopathologic and molecular perspective, similarities between PrBC and EOBC have been described at different clinicopathologic levels [12]. However, at least a subset PrBC is characterized by recurrent biological signatures resulting in immune tolerance, with reduced tumor-infiltrating lymphocytes (TILs) levels compared to EOBC [13,14,15,16]. This observation is consistent with the physiological modulation state of the maternal immune system to develop tolerance toward the semi-allogeneic fetus [17,18]. In this interaction, regulatory T cells (Tregs) and immune-checkpoint molecules such as programmed death-ligand 1 (PD-L1) play a crucial role [19,20]. The similarities between the mechanisms involved in maternal-fetal tolerance and tumor-host immunoediting suggest shared biological pathways [21]. However, whether the immune modulation that occurs during pregnancy has a significant impact on the development and progression of breast cancer has not been studied so far [22]. 

To date, no comprehensive data is available on the immune characteristics of PrBC as well as on the intrinsic composition of its tumor microenvironment (TME). We hypothesize that, if the clinical course of PrBC is driven by the immune milieu, this information could be used to assist clinical decision making and improve clinical trial design. In this study, we sought to characterize the TME of a large collection of PrBC, specifically focusing on the lymphocyte subpopulations, and to define new risk profiles based on the TME composition. 

## 2. Materials and Methods

### 2.1. Patients and Tissue Specimens

This study was approved by two local Ethical Committees under protocol numbers #620_2018bis and #UID3472. All patients included in this study were jointly diagnosed and managed at the European Institute of Oncology (IEO) and the Fondazione IRCCS Ca’ Granda—Ospedale Maggiore Policlinico (Milan, Italy) between February 2000 and November 2018. From our datasets, we retrieved a study group of PrBC and a control group of pregnancy unrelated EOBC. Exclusion criteria were personal or family history of breast cancer, documented cancer syndromes, and neoadjuvant treatments. All cases were reviewed, re-classified, and re-graded according to the latest World Health Organization (WHO) classification of breast tumors [23] and the Nottingham histologic grading system [24], respectively. Pathologic re-staging was performed following the 8th edition of the American Joint Committee on Cancer (AJCC) Cancer Staging Manual [25]. Breast cancer molecular subtypes were determined by ER, PgR, Ki67, and HER2 status following the St. Gallen International Expert Consensus recommendations [26]. Representative FFPE blocks were selected for tissue microarray construction as previously described, and used for all subsequent analyses [27]. For each case, the tumor core, its periphery (i.e., invasive front), and matched normal epithelial breast tissue (i.e., glandular tissue with at least one non-neoplastic terminal ductal-lobular unit adjacent to the neoplasm) were taken. 

### 2.2. Tumor-Infiltrating Lymphocytes Analysis

The evaluation of TILs was performed on 4 µm-thick hematoxylin and eosin-stained full-face sections at a ×200 magnification, based on the recommendations of the International TILs Working Group [28]. Specifically, TILs percentage was reported only for the stromal compartment as the area of stromal tissue occupied by mononuclear inflammatory cells (including lymphocytes and plasma cells) over the total intratumoral stromal area. TILs outside of the tumor border and around ductal carcinoma in situ (DCIS) and normal terminal duct-lobular units were not counted. The percentage of TILs was recorded both as a continuous value and as sub-categories: negative (<1%), low (1–20%), intermediate (21–50%), and high (>50%).

### 2.3. Immunohistochemical Analysis

Hormone receptors (HR) (i.e., estrogen receptor (ER), progesterone receptor (PgR)), Ki67, and HER2 status were updated to the breast biomarker reporting guidelines v1.4.1.1 published by the College of American Pathologists in November 2021 [29,30,31]. Then, lymphocyte subtyping was performed by immunohistochemistry (IHC) using antibodies against PD-L1 (clone 22C3), forkhead box P3 (FOXP3), CD4, and CD8 on an automated staining platform (i.e., Dako Omnis, Agilent, Santa Clara, CA, USA) on 4 µm-thick TMA sections, as previously described [32,33,34]. Both positive and negative controls were included in each run for each analysis. The presence and relative proportions of CD4-positive, CD8-positive, and FOXP3-positive cells within the TME were evaluated as the percentage of positive TILs [28,35]. Then, CD4 and FOXP3 were recorded as dichotomous variables based on the cut of the value of 1%, while CD8 was categorized as negative (<1%), low (1–30%), intermediate (31–50%), and high (>50%). Finally, PD-L1 analysis was based on the combined positive score (CPS), determined as the number of PD-L1 positive tumor cells, lymphocytes, and macrophages divided by the total number of viable tumor cells, multiplied by 100 [36,37,38]. Necrotic areas, as well as intraductal components, were excluded from the analysis. For the tumor cells, only the membrane staining, partial or complete, regardless of the staining intensity, was evaluated; for the immune cells, any membrane and/or cytoplasmic staining was included in the analysis. Any degree of staining intensity was considered for the scoring. According to the KEYNOTE-355 trial, the CPS was then sub-categorized using 10 as a cut-off value [38,39]. The methods and scoring systems employed are detailed in Supplementary Table S1.

### 2.4. Biostatistical Analysis

Categorical variables were summarized as counts and percentages, while for continuous variables means and standard deviations (SD) or median and Quartile 1 (Q1), Quartile 3 (Q3)) were used. Normal distributions of continuous variables were tested using the Shapiro–Wilk test. As a reference for the subtype prevalence in the general population of unselected breast cancer patients, clinical data were extracted from the MSK Cancer Cell 2018 dataset made available by The Cancer Genome Atlas Network (TCGA) at cBioPortal [40]. For statistical purposes, the frequency of MSK population was harmonized with the PrBC and EOBC through reducing the number of MSK population (*n* = 1752) to a 1:4 ratio randomly (*n* = 438). Differences in the baseline characteristics of PrBC patients versus the controls (i.e., EOBC or MSK) were assessed using Fisher’s exact or Chi-squared tests, and Wilcoxon rank-sum test or generalized linear models after testing for homoscedasticity (Levene test), for categorical and continuous variables, respectively. Likewise, the differences between patients who experienced progression and patients who did not, and between patients who died during follow-up and patients alive at the end of the follow-up were analyzed. The association with cancer progression or death during the follow-up was analyzed by survival analysis according to the Kaplan–Meier method and the log-rank test. Cox proportional hazard models were evaluated considering a stepwise selection procedure (*p*-value to entry into the model 0.15, *p*-value to stay 0.20) on variables associated with the outcome with a *p* ≤ 0.20 at the univariate level. The proportional hazard assumption was verified considering Schoenfeld’s residuals of the covariates. Adjusted hazard ratios and 95% confidence intervals were calculated. Two-tail *p*-values <0.05 were considered statistically significant. The analyses were performed using SAS statistical package, version 9.4 (SAS Institute Inc., Cary, NC, USA).

## 3. Results

### 3.1. Clinicopathological Features of PrBC

**Figure 1:**
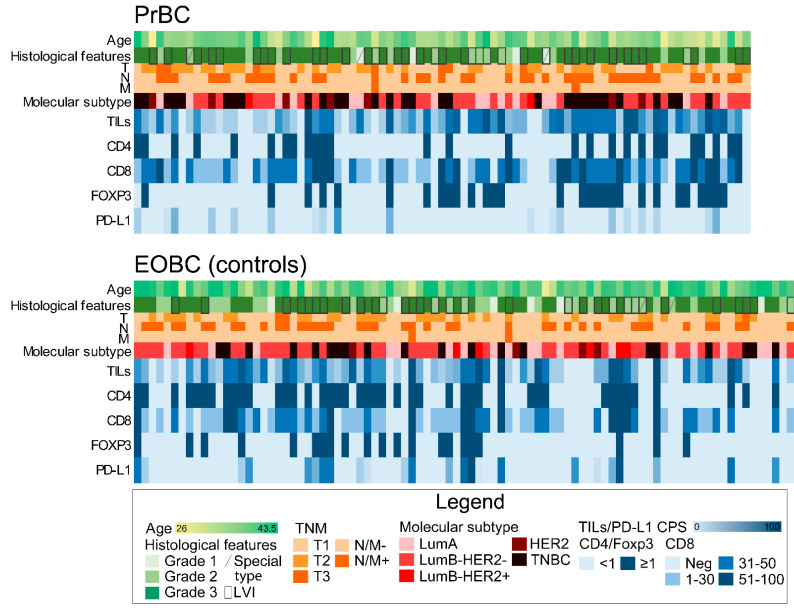
Heatmap illustrating selected clinicopathologic and immune-related features of breast cancers in pregnancy (PrBC) compared to the control group of early-onset breast cancers (EOBC) diagnosed in unpregnant women. Each column represents a patient and each row a parameter, color-coded according to the legend below. TILs, tumor-infiltrating lymphocytes; FOXP3, forkhead box P3; PD-L1, programmed death-ligand 1; LVI, lymph-vascular invasion; LumA, luminal A; LumB, luminal B; TNBC, triple-negative breast cancer; Neg, negative.

A total of 83 patients diagnosed with PrBC were included in this study (age range, 26–43 years; follow-up time, 1–247 months), including 13 cases that were part of a database from a previous publication of our group [13]. The control group consisted of 89 EOBCs diagnosed between 2004 and 2017 in non-pregnant patients (age range, 28–43 years; follow-up time of 1–203 months). Patients’ demographic and clinicopathologic characteristics for both the PrBC and EOBC groups are listed in Table 1 and detailed at a single-patient level in Figure 1. Detailed therapeutic data, including type and timing of systemic treatments, were available (Supplementary Table S2); treatment data of 46 EOBC were also accessible.

**Figure 2:**
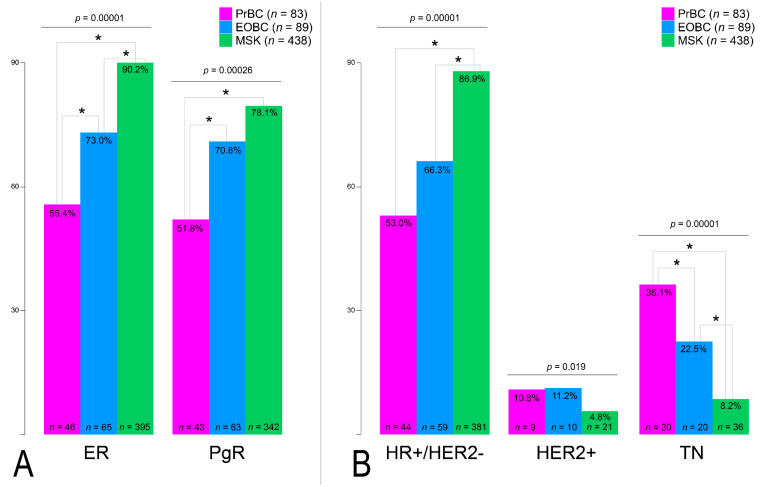
Analysis of biomarker status in the study population and controls. (**A**) Estrogen receptor (ER) and progesterone receptor (PgR) expression. (**B**) Prevalence of the three molecular clusters in the analyzed cases. HR, hormone receptors; TN, triple-negative breast cancer. Significant correlations among the different subset of patients (color-coded based on the legend on the right) are highlighted with a star (*).

The expression of HR was significantly lower in PrBC compared to EOBC (ER+ *n* = 46, 55.4% vs. *n* = 65, 73.0%; PgR+ *n* = 43, 51.8% vs. *n* = 63, 70.8%; *p* < 0.01), as shown in Table 1. This observation was also confirmed by the analysis of the MSK breast cancer dataset (*n* = 438; age range, 23–92 years; median, 52 years) that we employed as an external additional control group (ER+ *n* = 395, 90.2%; PgR+ *n* = 342, 78.1%), as shown in Figure 2A. Furthermore, the prevalence of the HR+/HER2– phenotype was significantly lower in PrBC (*p* < 0.01), with EOBC showing an intermediate frequency compared to MSK in all subgroups of breast cancers (*p* < 0.01), as shown in Figure 2B and Supplementary Table S2. These data confirm the high frequency of TNBC in PrBC, still after correcting the comparison for the age of the patients.

### 3.2. Increased CD8(+) TILs and Low/Null PD-L1 Expression in HR+/HER2– PrBC

**Figure 3:**
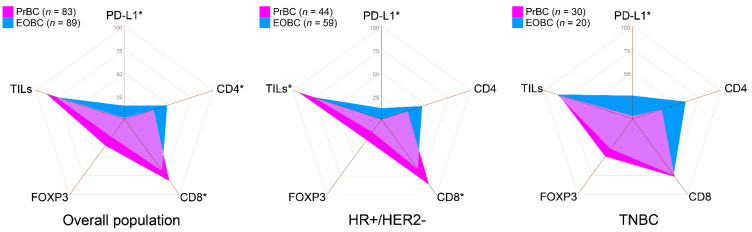
Immunograms showing the prevalence of patients with selected immune-related features in the study and control groups, according to the subtype. PrBC, breast cancer during pregnancy; EOBC, early-onset breast cancer; HR, hormone receptors; TNBC, triple-negative breast cancer; PD-L1, programmed death-ligand 1; TILs, tumor-infiltrating lymphocytes; FOXP3, forkhead box P3. Significant correlations among the different subset of patients (color-coded based on the legends) are highlighted with a star (*).

TILs were detected in 73 (88.0%) PrBC (low TILs *n* = 52, 71.2%; intermediate TILs *n* = 12, 16.4%; high TILs *n* = 9, 12.3%) and in 69 (77.5%) EOBC (low TILs *n* = 45, 65.2%; intermediate TILs *n* = 13, 18.8%; high TILs *n* = 11, 15.9%). Among all tumor subtypes, HR+/HER2– PrBC showed significantly higher TILs compared to HR+/HER2– EOBC (*n* = 41, 93.2% vs. 45, 76.3%; *p* = 0.022, Chi-square test), as shown in Table 2. The TILs phenotype was also significantly different between the two groups. In particular, CD8+ cells were more frequently detected in PrBC microenvironment (*n* = 68, 81.9% vs. *n* = 61, 68.5%; *p* = 0.043). This observation was confirmed in HR+/HER2– PrBC (*n* = 38, 86.4% vs. *n* = 39, 66.1%; *p* = 0.019) but not in TNBC (*n* = 23, 77.0% vs. *n* = 15, 75.0%; *p* = ns), as shown in Table 2 and Figure 3. 

**Figure 4:**
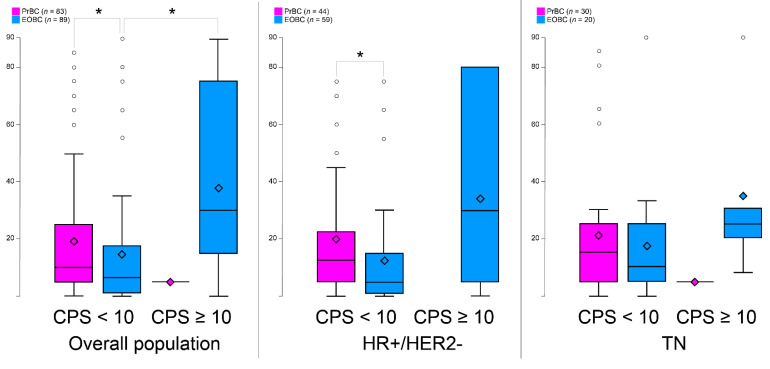
Tumor-infiltrating lymphocytes levels according to programmed death-ligand 1 (PD-L1) combined positive score (CPS) in the different molecular subtypes. PrBC, breast cancer during pregnancy; EOBC, early-onset breast cancer; HR, hormone receptors; TN, triple-negative breast cancer. Significant correlations among the different subset of patients (color-coded based on the legends) are highlighted with a star (*).

Not surprisingly, the amount of CD4+ cells mirrored that of CD8 +, where CD4+ cells were less present in PrBC than the EOBC (*p* = 0.035). No statistically significant differences were observed in the expression of FOXP3 in PrBC compared to the EOBC. The analysis of PD-L1 expression revealed that, despite the overall higher amount of TILs in PrBC, the majority of cases (*n* = 82, 98.8%) had CPS < 10, whereas 13 (14.6%) EOBC showed PD-L1 CPS ≥ 10 (*p* = 0.001) (Table 2). This significant difference was confirmed both in the HR+/HER2– (*p* = 0.018) and TNBC subsets (*p* = 0.021). We then quantified the percentage of TILs based on the PD-L1 status by CPS and TILs subpopulations and confirmed that PrBC with low PD-L1 showed a significant tendency towards harboring higher TILs both in the overall population (*p* = 0.037) and in the HR+/HER2– subtype (*p* = 0.015), as shown in Figure 4A–C and Supplementary Figure S1. These data suggest that PrBC, and in particular those belonging to the HR+/HER2– cluster, have a microenvironment enriched for cytotoxic T-cells in the absence of the negative immune-regulatory effect of PD-L1.

### 3.3. Clinical Outcome of PrBC Based on T-Cells Subpopulations

Overall, PrBC patients had a significantly higher risk of relapse (*n* = 36, 44.4%) and of disease-related death (*n* = 16, 19.8%), compared to EOBC (*n* = 6, 10.7%, *p* = 0.008; and *n* = 2, 3.6%, *p* = 0.006; respectively), as shown in Table 3. This increased risk was independent of the tumor subtype and HR/HER2 status. By stratifying patients based on the therapeutic regimens, for those who received chemotherapy, more PrBC experienced disease recurrence and death compared to EOBC (*n* = 31, 47% vs. *n* = 3, 9.7%; *p* = 0.0003 and, *n* = 16, 24.2% vs. *n* = 0; *p* = 0.003 respectively) (Supplementary Table S4).

Furthermore, a significant prognostic role of the TME characteristics was observed, whereby the presence of TILs, ranging from low to high, was protective in EOBC but it was related to relapses and death in PrBC (Table 3). The favorable prognostic role of TILs in EOBC was maintained even when the two cohorts were stratified both based on the receival of endocrine therapy and chemotherapy (*n* = 1, 7.1% vs. *n* = 18, 40.9%; *p* = 0.019 and *n* = 2, 6.9%, *n* = 26, 46.4%; *p* = 0.0002, respectively) (Supplementary Tables S4 and S5).

This observation was confirmed for each of the TIL subpopulations, including CD8+, CD4+, and FOXP3+ (Table 3). Considering the therapeutic regimens that were administered in each of the two cohorts, endocrine therapy was the most prevalent treatment in HR+HER2– PrBC patients (*n* = 42, 97.7% vs. *n* = 16, 51.6%; *p* = 0.0001). In terms of chemotherapy, a higher proportion of PrBC (*n* = 66, 83.5%) received such treatments compared to EOBCs (*n* = 31, 67.4%; *p* = 0.037) (Supplementary Table S3). For the therapeutic regimens and immune profiles, TILs presence was observed in a higher number of PrBC who were treated with endocrine therapy (*n* = 44, 62.9%) and in particular the CD8+ TILs (*n* = 40, 61.5) compared to EOBC patients (*n* = 14, 35.0%, *p* = 0.005 and *n* = 13, 36.1%, *p* = 0.012, respectively) treated with the same regimens. On the other hand, TILs differences in the two groups according to the cytotoxic treatment were not statistically significant (Supplementary Table S3). 

**Figure 5:**
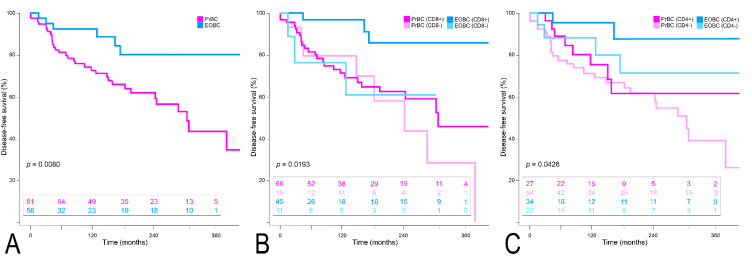
Disease-free survival analysis. (**A**) Overall population. (**B**) According to CD8+ TILs. (**C**) According to CD4+ TILs.

Survival analysis according to the Kaplan–Meier method confirmed the significantly worse DFS outcome of PrBC (*p* = 0.008), as displayed in Figure 5A. Even though CD8 and CD4 positivity were related to a better clinical course in the EOBC, the DFS in the PrBC remained worse independently of the biomarkers (*p* = 0.019, and *p* = 0.043), as shown in Figure 5B,C. Regarding the overall survival, although the clinical outcome was worse in the PrBC, the differences were not statistically significant neither in the overall comparison nor in different subtypes of PrBC compared to the EOBC (Supplementary Figure S2).

## 4. Discussion

In this study, we characterized the TME of a large set of PrBC and demonstrated that these tumors are immunologically and biologically distinct from conventional EOBC. First, we found that PrBC shows a lower frequency of HR expression and Luminal-like phenotype compared to both EOBC and the overall breast cancer population. Moreover, the frequency of immunologically “hot” tumors and the TILs phenotype is significantly different among the two groups of patients, where PrBC generally have a TILs profile similar to that of TN-EOBC but with less CD4 and PD-L1 expression. In particular, we were able to detect an increased CD8+ TILs and low/null PD-L1 expression in HR+/HER2– PrBC. In PD-L1 low/null cases, the PrBC showed a higher density of TILs compared to the EOBC. We also confirmed that PrBC patients have a significantly higher risk of relapse and death compared to EOBC, particularly for those patients with CD8+ TILs. 

Pregnancy is associated with hormonal changes that play a substantial part in shaping the immunological milieu during gestation for a successful term [41,42,43]. Sex hormones such as estrogen, may not only contribute to breast cancer development and progression, through their constant interaction with epithelial cells, but they may also play a regulatory role on the immune cells and TME [44]. In PrBC the rate of HR– breast cancer was significantly higher than in the age-matched EOBC and the overall population, independent of age range. Accordingly, HR status was influenced by both pregnancy status and age at the diagnosis of breast cancer. These findings are in line with previous studies [45,46] in which the ER– breast cancers were more frequently seen in PrBC (age <40) compared to EOBC [46]. Moreover, tumors during pregnancy and within a year of delivery were more frequently reported as triple-negative and HER2+ phenotype [45]. As the risk of developing breast cancer during pregnancy is lower than the post-delivery, it has been suggested that the higher frequency of HR– in PrBC could be related to the suppression of the HR+ tumors, rather than an increased risk of HR– tumor development. Moreover, the immunological changes induced during pregnancy could probably lead to tumor suppression [45]. CD8+ cells, one of the main components of adaptive immunity, are assumed to be delicately tempered during pregnancy [47]. Maternal CD8+ T cells with fetal specificity increase during pregnancy and persist after parturition [48,49]. Parallel to this, we found that the fraction of tumors with CD8+ TILs was significantly higher in PrBC than in the controls (*n* = 68, 81.9% vs. *n* = 61, 68.5%; *p* = 0.043), being mirrored by fewer cases with CD4+ TILs. Among all subgroups, the prevalence of tumors with documented TILs was significantly higher in HR+/HER2– PrBC (*n* = 41, 93.2% vs. 45, 76.3%; *p* = 0.022) compared to the HR+/HER2– EOBC. The results provided in the present work shed new light on those from a seminal paper on TILs in PrBC from our group, where a low prevalence of TILs was observed [13]. The cohorts of patients from the two studies, however, are different in the clinical setting, median age, and gestational age. Hence, the analyses for the 13 patients in common were consistent among the two studies (data available upon request). This observation militates in support of the critical need for multicentric efforts to unravel the complexity of these rare tumors, considering deeper analyses on additional clinical and molecular variables in a larger population of women. Regarding the TILs phenotype, the higher frequency of CD8+ cells was limited to the HR+/HER2– PrBC microenvironment (*n* = 38, 86.4% vs. *n* = 39, 66.1%; *p* = 0.019) compared to the HR+/HER2– EOBC. 

More PrBC than EOBC exhibited low/null expression of PD-L1 (*n* = 82, 98.8% and *n* = 76, 85.4%, respectively; *p* = 0.001), both in the HR+/HER2– and TNBC subsets. No differences were observed, however, for the HER2+ breast cancers, probably due to the small number of patients included in this category. At variance with our results, a previous study reported that PrBC had higher expression of both *PD-1* and *PD-L1* genes compared to the non-pregnant breast cancer population, suggesting that their high expression could induce immune suppression and hence result in aggressive tumor behavior [15]. It should be noted, however, that the current study was performed with an IHC assay, using a specific anti-PD-L1 monoclonal antibody and a specific threshold for a positive result. Furthermore, we analyzed the number of TILs after stratification for the low and high expression of PD-L1 (CPS <10 and ≥10, respectively). Accordingly, PrBC with low PD-L1 showed a significant tendency towards harboring higher TILs both in the overall population and in the HR+/HER2– subtype. This suggests that the TME of the PrBC, and in particular within the HR+/HER2– subtype, is enriched for cytotoxic T-cells in the absence or low expression of the negative immune-regulatory effect of PD-L1. Moreover, in EOBC PD-L1 expression was related to the amount of the immune cells while in PrBC PD-L1 expression was irrespective of the amount of the immune cells, and most of the tumors exhibited null/low expression of PD-L1. Given that tumors escape from immune surveillance is driven by diverse mechanisms according to their PD-L1 status and the presence or absence of TILs [50,51], we hypothesize that in EOBC the adaptive immune resistance is the major player, whereas in the PrBC with null/scarce PD-L1 expression other suppressors mechanisms may promote immune tolerance.

The prognosis of PrBC is still controversial, with some studies reporting a lower survival probability [52,53], and others a similar outcome as compared to EOBC [54,55,56]. In this study, a substantially higher proportion of PrBC patients experienced tumor progression and death (*n* = 36, 85.7%; and *n* = 16, 88.9%) compared to EOBC (*n* = 6, 14.3% and *n* = 2, 11.1%, respectively). This confirms previous studies reporting a poor outcome for PrBC, possibly due to the pregnancy-related physiological changes that may complicate and defer the diagnosis of breast cancer to later stages [7,11]. By further analysis, we saw that not only the presence of TILs but also individual TILs subpopulations (i.e., CD8, CD4, FOXP3) conferred a more aggressive clinical course of PrBC in comparison with EOBC. Importantly, TILs presence was seen yet in a greater proportion of PrBC patients compared to EOBC even when stratified based on the systemic treatments administered (i.e., endocrine therapy and chemotherapy). Additionally, even with this stratification, the protective role of TILs was confirmed in EOBC both in terms of disease recurrence and death. These findings need to be measured considering the notion that measurements of TILs density in routine clinical practice can be of prognostic value, especially for patients who receive adjuvant anthracycline [57]. In addition, all the three PrBC subtypes had a higher risk of relapse compared to the EOBC. PrBC with TILs and CD8+ cells had a higher risk of death compared to EOBC. It is of note that considering the low frequency of death among EOBC recruited for this study (*n*= 2) statistically significant findings were largely limited to the relapse incidence, with the relapse-free survival probability of PrBC being worse than for EOBC. Finally, CD4 and CD8 expression in PrBC did not correlate with DFS, opposite to their favorable prognostic value in EOBC.

This study has some limitations such as the use of tissue microarrays for the assessment of biomarkers expression, which although reliable, is not the most appropriate approach to overcome any possible intra-tumor heterogeneity. Among the breast cancer subtypes, the HER2+ population was under-represented in both PrBC (*n* = 9, 10.8%) and EOBC (10, 11.2%). Further studies including a larger number of patients with HER2+ breast cancer for assessment of TILs and TILs subtypes are warranted. Another intrinsic limitation is the systemic treatment difference between PrBC patients and controls because hormone therapy or anti-HER2 drugs are not administered during pregnancy and some PrBC patients, but not control patients had received weekly adjuvant anthracycline-based chemotherapy. Despite these limitations, our data provide novel insights into the composition of TME in PrBC and its potential correlation with patients’ clinical course.

## 5. Conclusions

The results of this study suggest that PrBC are enriched of TNBC phenotype and have specific patterns of TILs composition. Furthermore, PrBC is associated with worse clinical outcomes compared to EOBC, and this more aggressive clinical behavior is likely correlated with immunologic signatures. A routine assessment of TILs in these patients would be a valid addition to the pathology report that might help identify clinically relevant subsets of women with PrBC. Further studies on larger cohorts of PrBC patients could validate these findings and allow a deep analysis of TIL subpopulation functionality (e.g., cytokine expression, activating and inhibitory receptors expression) to better characterize TME.

## Figures and Tables

**Table 1:** Clinicopathological characteristics of the patients included in the study. PrBC, breast cancer during pregnancy; EOBC, early-onset breast cancers; standard deviation; SD, standard deviation; NST, no special type (aka ductal); LVI, lymph vascular invasion; ER, estrogen receptor; PgR, progesterone receptor; TNBC, triple-negative breast cancer. Significant correlations are highlighted with a star (*).

	PrBC	EOBC	*p*-Value
	(*n* = 83)	(*n* = 89)	
Age at diagnosis, years			<0.0001 *
Mean ± *SD*	35.1 ± 4.3	38.9 ± 3.7	
min, max	26, 43	28, 43	
Histological type, *n* (%)			0.137
NST	80 (96.4)	85 (95.5)	
Other	3 (3.6)	4 (4.5)	
LVI, *n* (%)	39 (47.0)	34 (38.2)	0.2441
T, *n* (%)			0.0898
T1	37 (44.6)	52 (58.4)	
T2	38 (45.8)	34 (38.2)	
T3/4	8 (9.6)	3 (3.4)	
N, *n* (%)			0.6582
N0	43 (51.8)	43 (52.4)	
N1	23 (27.7)	17 (20.7)	
N2	10 (12.1)	12 (14.6)	
N3	7 (8.4)	10 (12.2)	
M1, *n* (%)	2 (2.5)	0 (0.0)	0.2709
ER positive, *n* (%)	46 (55.4)	65 (73.0)	0.0158*
PgR positive, *n* (%)	43 (51.8)	63 (70.8)	0.0105*
Ki67 high, *n* (%)	65 (78.3)	66 (74.2)	0.5227
HER2 positive, *n* (%)	9 (10.8)	10 (11.2)	0.9346
Molecular subtype, *n* (%)			0.1445
Luminal-A	15 (18.1)	19 (21.4)	
Luminal-B (HER2–)	29 (34.9)	40 (44.9)	
Luminal-B (HER2+)	2 (2.4)	6 (6.7)	
HER2-type	7 (8.4)	4 (4.5)	
TNBC	30 (36.1)	20 (22.5)	
Subtypes, *n* (%)			0.1331
HR+/HER2–	44 (53.0)	59 (66.3)	0.0758
HER2+	9 (10.8)	10 (11.2)	0.9346
HR-/HER2–	30 (36.1)	20 (22.5)	0.0485 *

**Table 2:** Relative prevalence of TILs subpopulation in the tumor stroma and PD-L1 expression in breast cancer subtypes. PrBC, breast cancer during pregnancy; EOBC, early-onset breast cancers; HR, hormone receptors; TILs, tumor-infiltrating lymphocytes; FOXP3, forkhead box P3; PD-L1, programmed death-ligand 1; CPS, combined positive score. Significant correlations are highlighted with a star (*).

	Total Population			HR+/HER2–			HR-/HER2–		
	PrBC	EOBC	*p*-Value	PrBC	EOBC	*p*-Value	PrBC	EOBC	*p*-Value
	(*n* = 83)	(*n* = 89)		(*n* = 44)	(*n* = 59)		(*n* = 30)	(*n* = 20)	
**TILs, *n* (%)**									
Absence	10 (12.0)	20 (22.5)	0.0718	3 (6.8)	14 (23.7)	0.0222 *	5 (16.7)	3 (15.0)	0.8749
Presence	73 (88.0)	69 (77.5)		41 (93.2)	45 (76.3)		25 (83.3)	17 (85.0)	
Low	52 (71.2)	45 (65.2)		30 (73.2)	33 (73.3)		16 (64.0)	9 (52.9)	
Intermediate	12 (16.4)	13 (18.8)		6 (14.6)	6 (13.3)		5 (20.0)	6 (35.3)	
High	9 (12.3)	11 (15.9)		5 (12.2)	6 (13.3)		4 (16.0)	2 (11.8)	
**CD8, *n* (%)**									
Absence	15 (18.1)	28 (31.5)	0.0427 *	6 (13.6)	20 (33.9)	0.0192 *	7 (23.0)	5 (25.0)	0.8925
Presence	68 (81.9)	61 (68.5)		38 (86.4)	39 (66.1)		23 (77.0)	15 (75.0)	
Low	23 (27.7)	30 (33.7)		16 (42.1)	20 (51.3)		5 (21.7)	7 (46.7)	
Intermediate	29 (34.9)	19 (21.3)		14 (36.8)	14 (35.9)		11 (47.8)	3 (20.0)	
High	16 (19.3)	12 (13.5)		8 (21.1)	5 (12.8)		7 (30.4)	5 (33.3)	
**CD4, *n* (%)**									
Absence	56 (67.5)	46 (51.7)	0.0352 *	31 (70.5)	32 (54.2)	0.0948	20 (66.7)	8 (40.0)	0.0627
Presence	27 (32.5)	43 (48.3)		13 (29.5)	27 (45.8)		10 (33.3)	12 (60.0)	
**FOXP3, *n* (%)**									
Absence	54 (65.1)	68 (76.4)	0.1016	32 (72.7)	49 (83.1)	0.206	15 (50.0)	12 (60.0)	0.487
Presence	29 (34.9)	21 (23.6)		12 (27.3)	10 (16.9)		15 (50.0)	8 (40.0)	
**PD-L1 CPS, *n* (%)**									
<10	82 (98.8)	76 (85.4)	0.0013 *	44 (100)	52 (88.1)	0.0179 *	29 (96.7)	15 (75.0)	0.0209 *
≥10	1 (1.2)	13 (14.6)		0	7 (11.9)		1 (3.3)	5 (25.0)	

**Table 3:** Disease progression and patients’ death status in the study and control groups. PrBC, breast cancer during pregnancy; EOBC, early-onset breast cancers; HR, hormone receptors; TILs, tumor-infiltrating lymphocytes; FOXP3, forkhead box P3; PD-L1, programmed death-ligand 1; CPS, combined positive score. Significant correlations are highlighted with a star (*).

	Disease Recurrence						Died of Disease			
	PrBC		EOBC		*p*-Value	PrBC		EOBC		*p*-Value
	Yes	No	Yes	No		Yes	No	Yes	No	
**Cases with FU, *n* (%)**	36 (44.4)	45 (55.6)	6 (10.7)	50 (89.3)	0.0080 *	16 (19.8)	65 (80.2)	2 (3.6)	54 (96.4)	0.0059 *
HR+HER2–	17 (38.6)	27 (61.4)	5 (13.5)	32 (86.5)	0.0113 *	6 (13.6)	38 (86.4)	1 (2.7)	36 (97.3)	0.0811
HER2+	5 (55.6)	4 (44.4)	0	6 (100)	0.0253 *	2 (20.0)	8 (80.0)	0	6 (100)	0.2416
HR-/HER2–	14 (50.0)	14 (50.0)	1 (7.7)	12 (92.3)	0.0089 *	8 (28.6)	20 (71.4)	1 (7.7)	12 (92.3)	0.1328
**Presence of TILs, *n* (%)**	31 (43.7)	40 (56.3)	3 (6.1)	46 (93.9)	<0.0001 *	13 (18.3)	58 (81.7)	0	49 (100)	0.0015 *
Low	23 (46.0)	27 (44.0)	3 (9.4)	29 (90.6)	0.0005 *	10 (20.0)	40 (80.0)	0	32 (100)	0.0069 *
Intermediate	6 (50.0)	6 (50.0)	0	9 (100)	0.0121 *	2 (16.6)	10 (83.3)	0	9 (100)	0.1978
High	2 (22.2)	7 (77.8)	0	8 (100)	0.1557	1 (11.1)	8 (88.9)	0	8 (100)	0.3312
**CD8 TILs, *n* (%)**	28 (42.4)	38 (57.6)	3 (6.7)	42 (93.3)	<0.0001 *	13 (19.7)	53 (80.3)	0	45 (100)	0.0015 *
Low	14 (63.6)	8 (36.4)	2 (8.7)	21(91.3)	0.0001 *	8 (36.4)	14 (63.6)	0	23 (100)	0.0014 *
Intermediate	7 (25.0)	21 (75.0)	1 (8.3)	11(91.7)	0.2272	3 (10.7)	25 (89.3)	0	12 (100)	0.2384
High	7 (43.7)	9 (56.3)	0	10 (100)	0.0144 *	2 (12.5)	14 (87.5)	0	10 (100)	0.2445
**CD4 TILs, *n* (%)**	9 (33.3)	18 (66.7)	2 (5.6)	32 (94.4)	0.0056 *	2 (7.4)	25 (92.6)	0	34 (100)	0.1066
**FOXP3, *n* (%)**	9 (33.3)	18 (66.7)	0	21 (100)	0.0033 *	3 (11.1)	24 (88.9)	0	21 (100)	0.1146
**PD-L1 CPS ≥ 10, *n* (%)**	-	-	0	9 (100)	-	-	-	0	9 (100)	-

## Data Availability

Links to publicly archived datasets analyzed or generated during the study: https://www.cbioportal.org/ (accessed on 14 January 2022).

## Supplementary Materials

The following supporting information can be downloaded at: https://www.mdpi.com/article/10.3390/cells11152286/s1, Figure S1_1–_6: Association between stromal tumor-infiltrating lymphocytes (TILs) density and TILs subpopulations; Figure S2a_1–a_5: Overall survival analysis in PrBC and EOBC; Figure S2b_1–b_3: Disease-free survival analysis in PrBC and EOBC; Table S1: List of antibodies used for immunohistochemical analyses; Table S2. Analysis of biomarker status in the study population and controls. Table S3. Therapeutic data of the PrBC and the EOBC. Table S4. Disease progression and patients’ death status both in PrBC and EOBC based on the administration of chemotherapy. Table S5. Disease progression and patients’ death status in the study and control groups based on the administration of endocrine therapy.
