# Supplementary material for: Breast Cancer during Pregnancy as a Special Type of Early-Onset Breast Cancer: Analysis of the Tumor Immune Microenvironment and Risk Profiles
Source: Cells. 2022 Jul 24;11(15):2286. doi: 10.3390/cells11152286 (PMC9332147; doi:10.3390/cells11152286)
Supplement: Supplementary file 1 [file cells-11-02286-s001.zip › cells-1749765-supplementary-tables.pdf]

cells MDPI

# Supplementary Tables

**Table S1.** List of antibodies used for immunohistochemical analyses. The analyses were performed on a Dako Omnis staining platform using the polymer-based EnVision FLEX antigen retrieval system. PD-L1, programmed death-ligand 1; RTU, ready to use.

| Marker | Clone | Dilution | Antigen Retrieval | Scoring |
|---|---|---|---|---|
| ER | EP1 | RTU | High pH, 20' | ASCO/CAP and St Gallen guidelines; positive if ≥1% of tumor cell nuclei are immunoreactive. |
| PgR | PgR 636 | 1:100 | High pH, 30′ | ASCO/CAP and St Gallen guidelines; positive if ≥1% of tumor cell nuclei are immunoreactive. |
| Ki-67 | MIB1 | RTU | High pH, 30′ | International Ki67 in Breast Cancer Working Group; high if ≥30% of tumor cell nuclei are immunoreactive |
| HER2 | Polyclonal | 1:400 | Low pH, 30' | ASCO/CAP guidelines; 3+ if complete membrane staining that is intense and >10% of tumor cells; 2+ if weak to moderate complete membrane staining in >10% of tumor cells or complete membrane staining that is intense but within ≤10% of tumor cells; 1+ if incomplete membrane staining that is faint/barely perceptible and within >10% of tumor cells; 0 if no staining observed or membrane stating that is incomplete and is faint/barely perceptible and within ≤10% of tumor cells. |
| TILs | n/a | n/a | n/a | |
| CD4 | 4B12 | RTU | Low pH, 30' | Negative if complete loss of membrane staining within the lymphocytes. |
| CD8 | C8/144B | 1:50 | Low pH, 30' | Negative if complete loss of membrane staining within the lymphocytes; low, 1–30%; intermediate, 31–50%; high, >50%. |
| FOXP3 | Polyclonal | 1:200 | Low pH, 30' | Negative if complete loss of membrane staining within the lymphocytes. |
| PD-L1 | 22C3 | RTU | Low pH, 30' | Combined positive score (CPS), i.e. total number of PD-L1+ tumor cells, lymphocytes, and macrophages divided by the total number of viable tumor cells, multiplied by 100. |

**Table S2.** Analysis of biomarker status in the study population and controls. Estrogen receptor (ER) and progesterone receptor (PR) expression. HR, hormone receptors. Significant correlations among the different subsets of patients are highlighted with a star (*).

| | **PrBC (*n* = 83)** | **EOBC (*n* = 89)** | **MSK (*n* = 438)** | ***p*-value** |
|---|---|---|---|---|
| ER+, *n* (%) | 46 (55.4) | 65 (73.0) | 395 (90.2) | <0.0001* |
| PgR+, *n* (%) | 43 (51.8) | 63 (70.8) | 342 (78.1) | <0.0001* |
| Subtypes, *n* (%) | | | | |
| HR+/HER2- | 44 (53.0) | 59 (66.3) | 381 (86.9) | <0.0001* |
| HER2+ | 9 (10.8) | 10 (11.2) | 21 (4.8) | 0.019* |
| HR-/HER2- | 30 (36.1) | 20 (22.5) | 36 (8.2) | <0.0001* |

**Table S3.** Therapeutic data of the PrBC and the EOBC. PrBC, breast cancer during pregnancy; EOBC, early-onset breast cancers; HR, hormone receptors; TILs, tumor-infiltrating lymphocytes; FOXP3, forkhead box P3; PD-L1, programmed death-ligand 1; CPS, combined positive score. Significant correlations are highlighted with a star (*).

| | **Endocrine Therapy** | | | | | **Chemotherapy** | | | | |
|---|---|---|---|---|---|---|---|---|---|---|
| | **PrBC** | | **EOBC** | | ***p*-Value** | **PrBC** | | **EOBC** | | ***p*-Value** |
| | **Yes** | **No** | **Yes** | **No** | | **Yes** | **No** | **Yes** | **No** | |
| **All cases with FU, *n* (%)** | 49 (62.0) | 30 (38.0) | 18 (39.1) | 28 (60.9) | 0.0133* | 66 (83.5) | 13 (16.5) | 31 (67.4) | 15 (32.6) | 0.0367* |
| HR+HER2- | 42 (97.7) | 1 (2.3) | 16 (51.6) | 15 (48.4) | 0.0001* | 30 (71.4) | 12 (28.6) | 19 (61.3) | 12 (38.7) | 0.3620 |
| HER2+ | 2 (22.2) | 7 (77.8) | 1 (25.0) | 3 (75.0) | 0.9126 | 9 (100.0) | 0 | 3 (75.0) | 1 (25.0) | 0.2416 |
| HR-/HER2- | 5 (18.5) | 22 (81.5) | 1 (9.1) | 10 (90.9) | 0.4698 | 27 (96.4) | 1 (3.6) | 9 (81.8) | 2 (18.2) | 0.1233 |
| **Presence of TILs, *n* (%)** | 44 (62.9) | 26 (37.1) | 14 (35.0) | 26 (65.0) | 0.0048* | 56 (81.2) | 13 (18.8) | 29 (72.5) | 11 (27.5) | 0.2929 |
| Low | 32 (65.3) | 17 (34.7) | 10 (37.0) | 17 (63.0) | 0.0176* | 39 (81.3) | 9 (18.7) | 20 (74.1) | 7 (25.9) | 0.4625 |
| Intermediate | 7 (58.3) | 5 (41.7) | 2 (25.0) | 6 (75.0) | 0.1421 | 11 (91.7) | 1 (8.3) | 6 (75.0) | 2 (25.0) | 0.3064 |
| High | 5 (55.6) | 4 (44.4) | 2 (40.0) | 3 (60.0) | 0.5769 | 6 (66.7) | 3 (33.3) | 3 (60.0) | 2 (40.0) | 0.8030 |
| **CD8 TILs, *n* (%)** | 40 (61.5) | 25 (38.5) | 13 (36.1) | 23 (63.9) | 0.0125* | 55 (85.9) | 9 (14.1) | 26 (72.2) | 10 (27.8) | 0.0933 |
| Low | 15 (68.2) | 7 (31.8) | 9 (45.0) | 11 (55.0) | 0.1294 | 19 (86.4) | 3 (13.6) | 13 (65.0) | 7 (35.0) | 0.1044 |
| Intermediate | 16 (59.3) | 11 (40.7) | 2 (22.2) | 7 (77.8) | 0.0542 | 20 (76.9) | 6 (23.1) | 8 (88.9) | 1 (11.1) | 0.4339 |
| High | 9 (56.3) | 7 (43.7) | 2 (28.6) | 5 (71.4) | 0.2214 | 16 (100.0) | 0 | 5 (71.4) | 2 (28.6) | 0.2445 |
| **CD4 TILs, *n* (%)** | 15 (55.6) | 12 (44.4) | 11 (36.7) | 19 (63.3) | 0.1528 | 26 (96.3) | 1 (3.7) | 23 (76.7) | 7 (23.3) | 0.0331* |
| **FOXP3, *n* (%)** | 15 (55.6) | 12 (44.4) | 7 (41.2) | 10 (58.8) | 0.3529 | 23 (85.2) | 4 (14.8) | 10 (58.8) | 7 (41.2) | 0.0492* |
| **PD-L1 CPS ≥10, *n* (%)** | - | - | 3 (37.5) | 5 (62.5) | - | - | - | 5 (62.5) | 3 (37.5) | - |

**Table S4.** Disease progression and patients' death status both in PrBC and EOBC based on the administration of chemotherapy. PrBC, breast cancer during pregnancy; EOBC, early-onset breast cancers; HR, hormone receptors; TILs, tumor-infiltrating lymphocytes; FOXP3, forkhead box P3; PD-L1, programmed death-ligand 1; CPS, combined positive score. Significant correlations are highlighted with a star (*).

| | **Chemotherapy** | | | | | | | | | |
|---|---|---|---|---|---|---|---|---|---|---|
| | **Disease Recurrence** | | | | | **Died of Disease** | | | | |
| | **PrBC** | | **EOBC** | | ***p*-Value** | **PrBC** | | **EOBC** | | ***p*-Value** |
| | **Yes** | **No** | **Yes** | **No** | | **Yes** | **No** | **Yes** | **No** | |
| **All cases with FU, *n* (%)** | 31 (47.0) | 35 (53.0) | 3 (9.7) | 28 (90.3) | 0.0003* | 16 (24.2) | 50 (75.8) | 0 | 31 (100) | 0.0027* |
| HR+HER2- | 13 (43.3) | 17 (56.7) | 3 (15.8) | 16 (84.2) | 0.0451* | 6 (20.0) | 24 (80.0) | 0 | 19 (100) | 0.0374* |
| HER2+ | 5 (55.6) | 4 (44.4) | 0 | 3 (100) | 0.0909 | 2 (22.2) | 7 (77.8) | 0 | 3 (100) | 0.3710 |
| HR-/HER2- | 13 (48.1) | 14 (51.9) | 0 | 9 (100) | 0.0092* | 8 (29.6) | 19 (70.4) | 0 | 9 (100) | 0.0641 |
| **Presence of TILs, *n* (%)** | 26 (46.4) | 30 (53.6) | 2 (6.9) | 27 (93.1) | 0.0002* | 13 (23.2) | 43 (76.8) | 0 | 29 (100) | 0.0048* |
| Low | 19 (48.7) | 20 (51.3) | 2 (10.0) | 18 (90.0) | 0.0032* | 10 (25.6) | 29 (74.4) | 0 | 20 (100) | 0.0129* |
| Intermediate | 5 (45.4) | 6 (54.5) | 0 | 6 (100) | 0.0493* | 2 (18.2) | 9 (81.8) | 0 | 6 (100) | 0.2662 |
| High | 2 (33.3) | 4 (66.6) | 0 | 3 (100) | 0.2568 | 1 (16.6) | 5 (83.3) | 0 | 3 (100) | 0.4532 |
| **CD8 TILs, *n* (%)** | 16 (40.0) | 24 (60.0) | 2 (7.7) | 24 (92.3) | 0.0039* | 8 (20.0) | 32 (80.0) | 0 | 26 (100) | 0.0149* |
| Low | 13 (68.4) | 6 (31.6) | 1 (7.7) | 12 (92.3) | 0.0007* | 8 (42.1) | 11 (57.9) | 0 | 13 (100) | 0.0069* |
| Intermediate | 6 (30.0) | 14 (70.0) | 1 (12.5) | 7 (87.5) | 0.3339 | 3 (15.0) | 17 (85.0) | 0 | 8 (100) | 0.2463 |
| High | 7 (43.7) | 9 (56.3) | 0 | 5 (100) | 0.0701 | 2 (12.5) | 14 (87.5) | 0 | 5 (100) | 0.4058 |
| **CD4 TILs, *n* (%)** | 9 (34.6) | 17 (65.4) | 2 (8.7) | 21 (91.3) | 0.0299* | 2 (7.7) | 24 (92.3) | 0 | 23 (100) | 0.1744 |
| **FOXP3, *n* (%)** | 9 (39.1) | 14 (60.9) | 0 | 10 (100) | 0.0204* | 3 (13.0) | 20 (87.0) | 0 | 10 (100) | 0.2309 |

| Chemotherapy | | | | | | | | | | |
|---|---|---|---|---|---|---|---|---|---|---|
| | **Disease Recurrence** | | | | | **Died of Disease** | | | | |
| | **PrBC** | | **EOBC** | | ***p*-Value** | **PrBC** | | **EOBC** | | ***p*-Value** |
| | **Yes** | **No** | **Yes** | **No** | | **Yes** | **No** | **Yes** | **No** | |
| **PD-L1 CPS ≥10, *n* (%)** | 1 (33.3) | 2 (66.6) | 0 | 5 (100) | 0.1675 | 0 | 3 (100) | 0 | 5 (100) | - |

**Table S5.** Disease progression and patients' death status in the study and control groups based on the administration of endocrine therapy. PrBC, breast cancer during pregnancy; EOBC, early-onset breast cancers; HR, hormone receptors; TILs, tumor-infiltrating lymphocytes; FOXP3, forkhead box P3; PD-L1, programmed death-ligand 1; CPS, combined positive score. Significant correlations are highlighted with a star (*).

| Endocrine Therapy | | | | | | | | | | |
|---|---|---|---|---|---|---|---|---|---|---|
| | **Disease Recurrence** | | | | | **Died of Disease** | | | | |
| | **PrBC** | | **EOBC** | | ***p*-Value** | **PrBC** | | **EOBC** | | ***p*-Value** |
| | **Yes** | **No** | **Yes** | **No** | | **Yes** | **No** | **Yes** | **No** | |
| **All cases with FU, *n* (%)** | 20 (40.8) | 29 (59.2) | 3 (16.6) | 15 (83.3) | 0.0649 | 10 (20.4) | 39 (79.6) | 1 (5.6) | 17 (94.6) | 0.1457 |
| HR+HER2- | 16 (38.1) | 26 (61.9) | 3 (18.8) | 13 (81.2) | 0.1606 | 6 (14.3) | 36 (85.7) | 1 (6.3) | 15 (93.7) | 0.4011 |
| HER2+ | 1 (50.0) | 1 (50.0) | 0 | 1 (100) | 0.3864 | 1 (100) | 1 | 0 | 1 (100) | 0.3864 |
| HR-/HER2- | 3 (60.0) | 2 (40.0) | 0 | 1 (100) | 0.2733 | 3 (60.0) | 2 (40.0) | 0 | 1 (100) | 0.2733 |
| **Presence of TILs, *n* (%)** | 18 (40.9) | 26 (59.1) | 1 (7.1) | 13 (92.9) | 0.0190* | 8 (18.2) | 36 (81.8) | 0 | 14 (100) | 0.0857 |
| Low | 13 (40.6) | 19 (59.4) | 1 (10.0) | 9 (90.0) | 0.0729 | 6 (18.8) | 26 (81.2) | 0 | 10 (100) | 0.1391 |
| Intermediate | 4 (57.1) | 3 (42.9) | 0 | 2 (100) | 0.1515 | 1 (14.3) | 6 (85.7) | 0 | 2 (100) | 0.5707 |
| High | 1 (20.0) | 4 (80.0) | 0 | 2 (100) | 0.4945 | 1 (20.0) | 4 (80.0) | 0 | 2 (100) | 0.4945 |
| **CD8 TILs, *n* (%)** | 16 (40.0) | 24 (60.0) | 1 (7.7) | 12 (92.3) | 0.0301* | 8 (20.0) | 32 (80.0) | 0 | 13 (100) | 0.0801 |
| Low | 9 (60.0) | 6 (40.0) | 1 (11.1) | 8 (88.9) | 0.0186* | 5 (33.3) | 10 (66.6) | 0 | 9 (100) | 0.0515 |
| Intermediate | 4 (25.0) | 12 (75.0) | 0 | 2 (100) | 0.4226 | 2 (12.5) | 14 (87.5) | 0 | 2 (100) | 0.5958 |
| High | 3 (33.3) | 6 (66.6) | 0 | 2 (100) | 0.3383 | 1 (11.1) | 8 (88.9) | 0 | 2 (100) | 0.6210 |
| **CD4 TILs, *n* (%)** | 4 (26.6) | 11 (73.3) | 1 (9.1) | 10 (90.9) | 0.2612 | 1 (6.66) | 14 (93.3) | 0 | 11 (100) | 0.3825 |
| **FOXP3, *n* (%)** | 6 (40.0) | 9 (60.0) | 0 | 7 (100) | 0.0497* | 3 (20.0) | 12 (80.0) | 0 | 7 (100) | 0.2029 |
| **PD-L1 CPS ≥10, *n* (%)** | 0 | 2 (100) | 0 | 4 (100) | - | 0 | 2 (100) | 0 | 4 (100) | - |
