# Supplementary figures and images for: Breast Cancer during Pregnancy as a Special Type of Early-Onset Breast Cancer: Analysis of the Tumor Immune Microenvironment and Risk Profiles
Source: Cells. 2022 Jul 24;11(15):2286. doi: 10.3390/cells11152286 (PMC9332147; doi:10.3390/cells11152286)

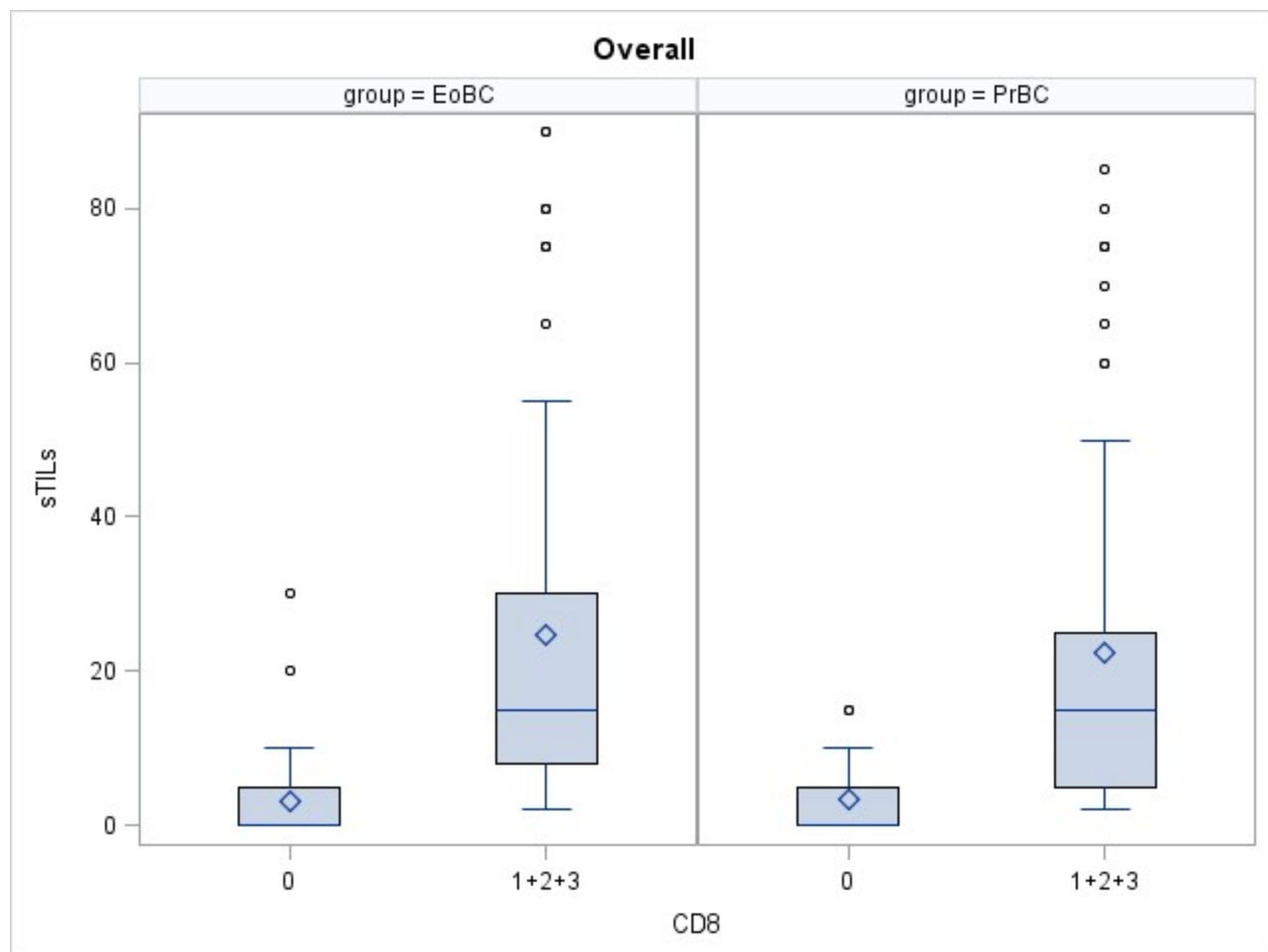

Supplement: Supplementary file 1 [file cells-11-02286-s001.zip › Supplementary Figure S1_1.pdf]

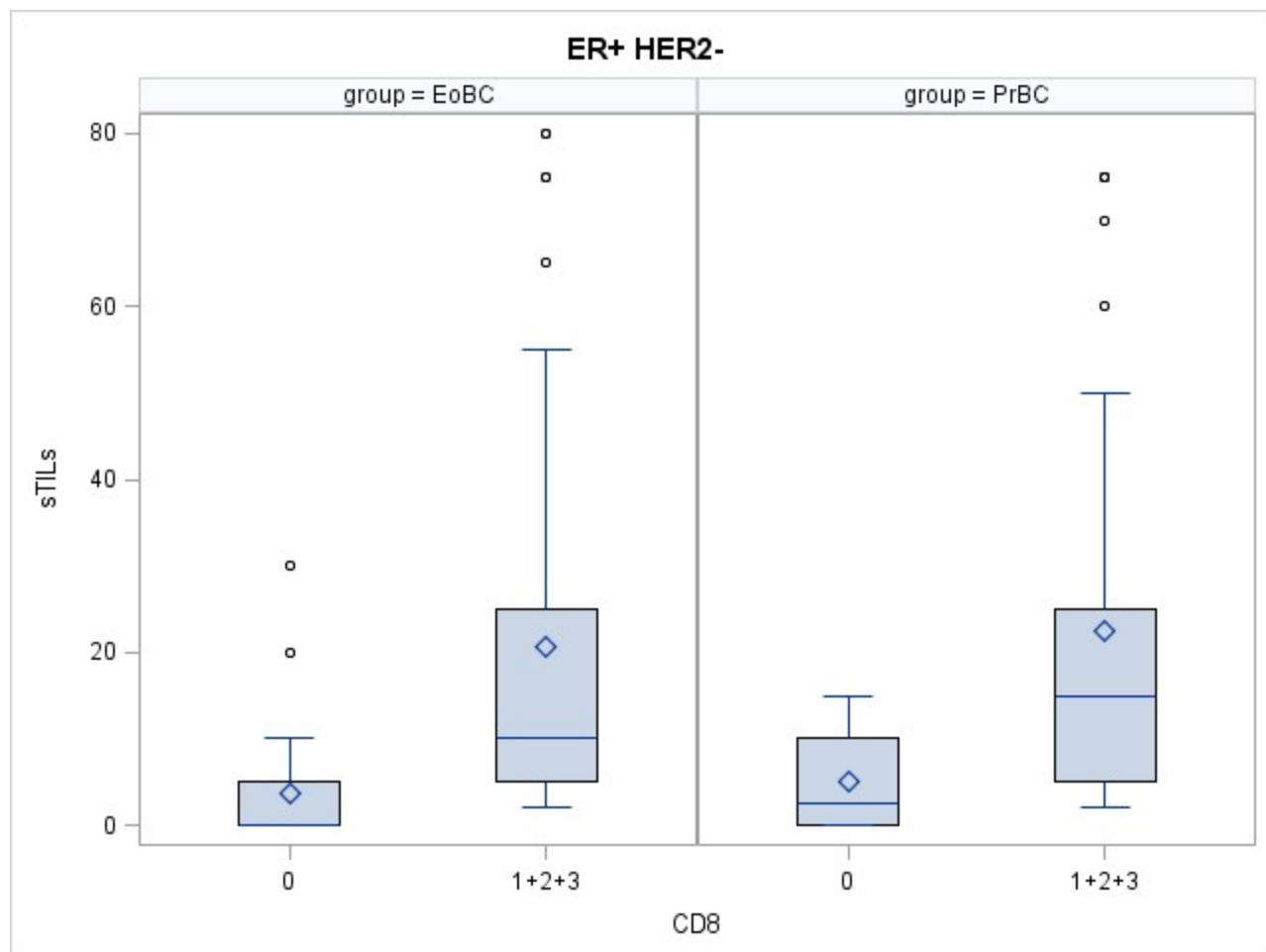

Supplement: Supplementary file 1 [file cells-11-02286-s001.zip › Supplementary Figure S1_2.pdf]

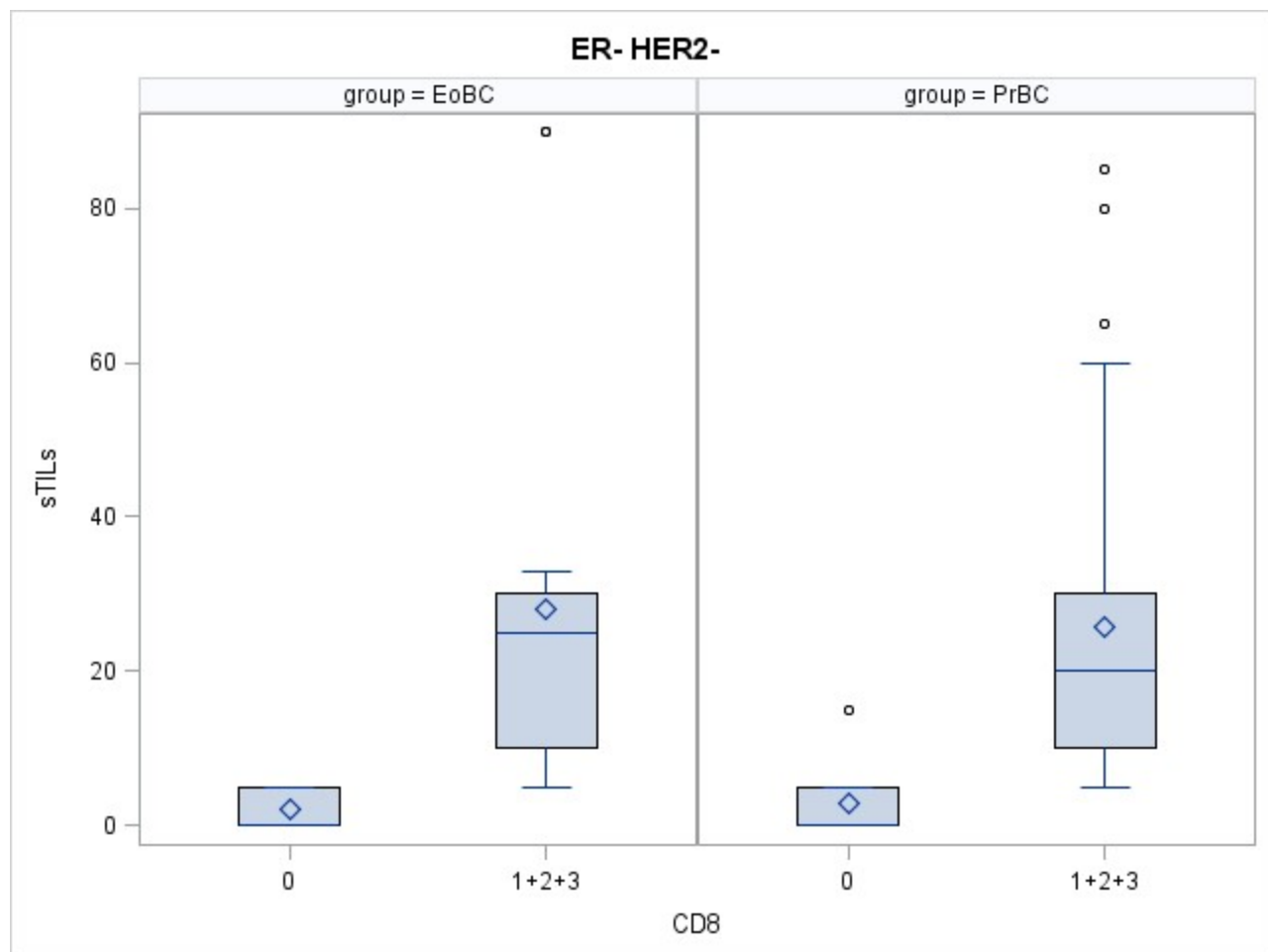

Supplement: Supplementary file 1 [file cells-11-02286-s001.zip › Supplementary Figure S1_3.pdf]

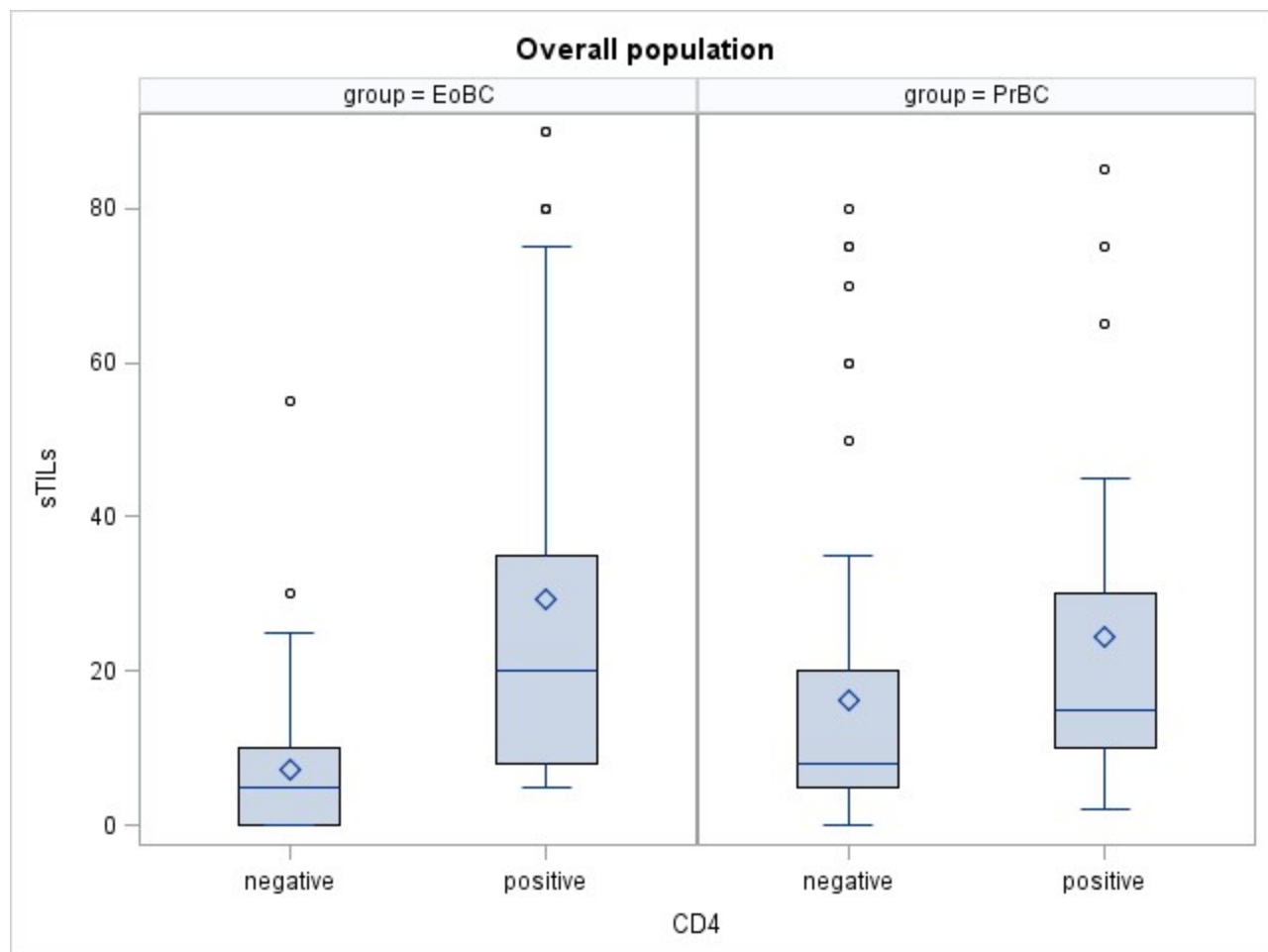

Supplement: Supplementary file 1 [file cells-11-02286-s001.zip › Supplementary Figure S1_4.pdf]

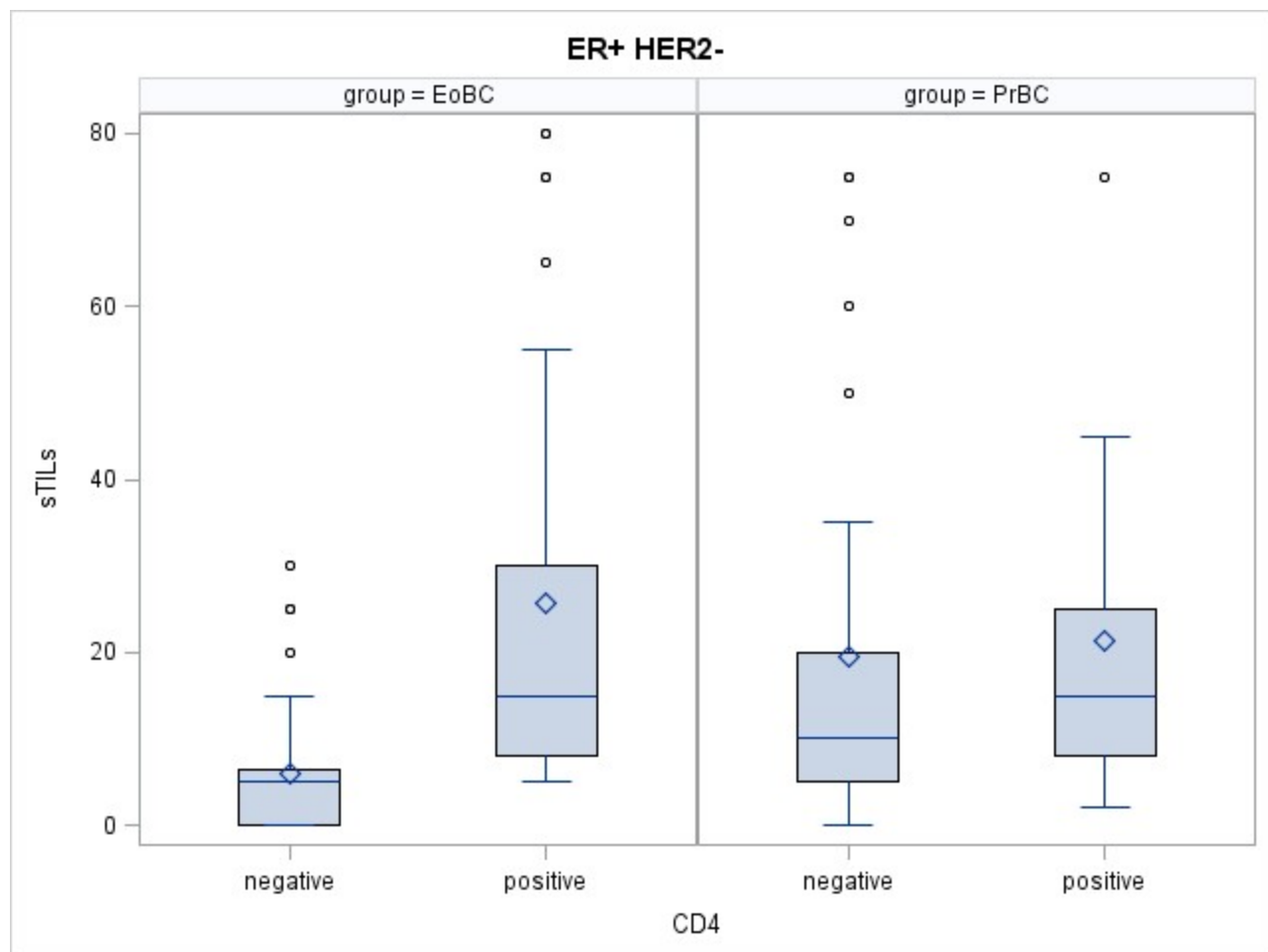

Supplement: Supplementary file 1 [file cells-11-02286-s001.zip › Supplementary Figure S1_5.pdf]

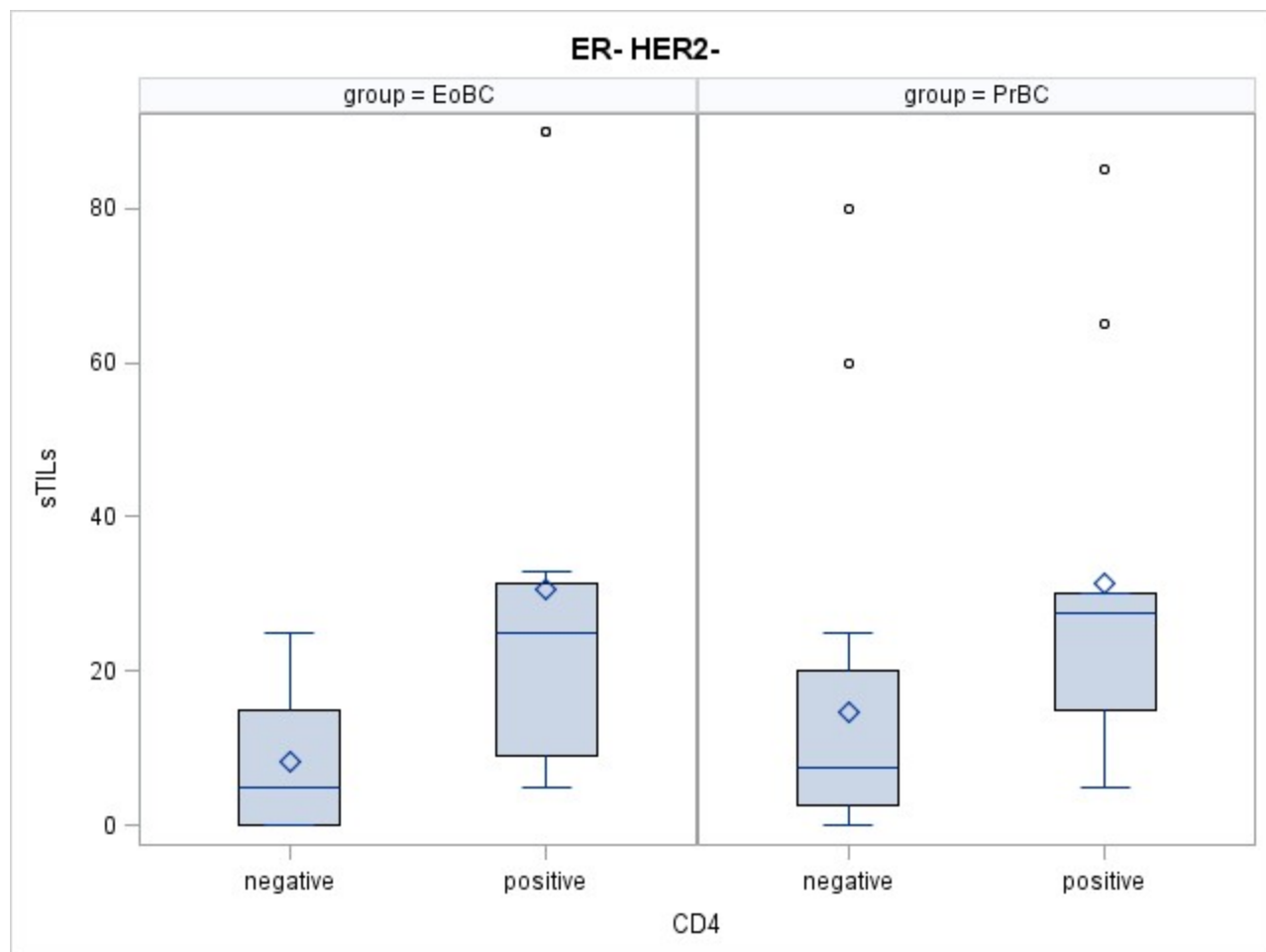

Supplement: Supplementary file 1 [file cells-11-02286-s001.zip › Supplementary Figure S1_6.pdf]

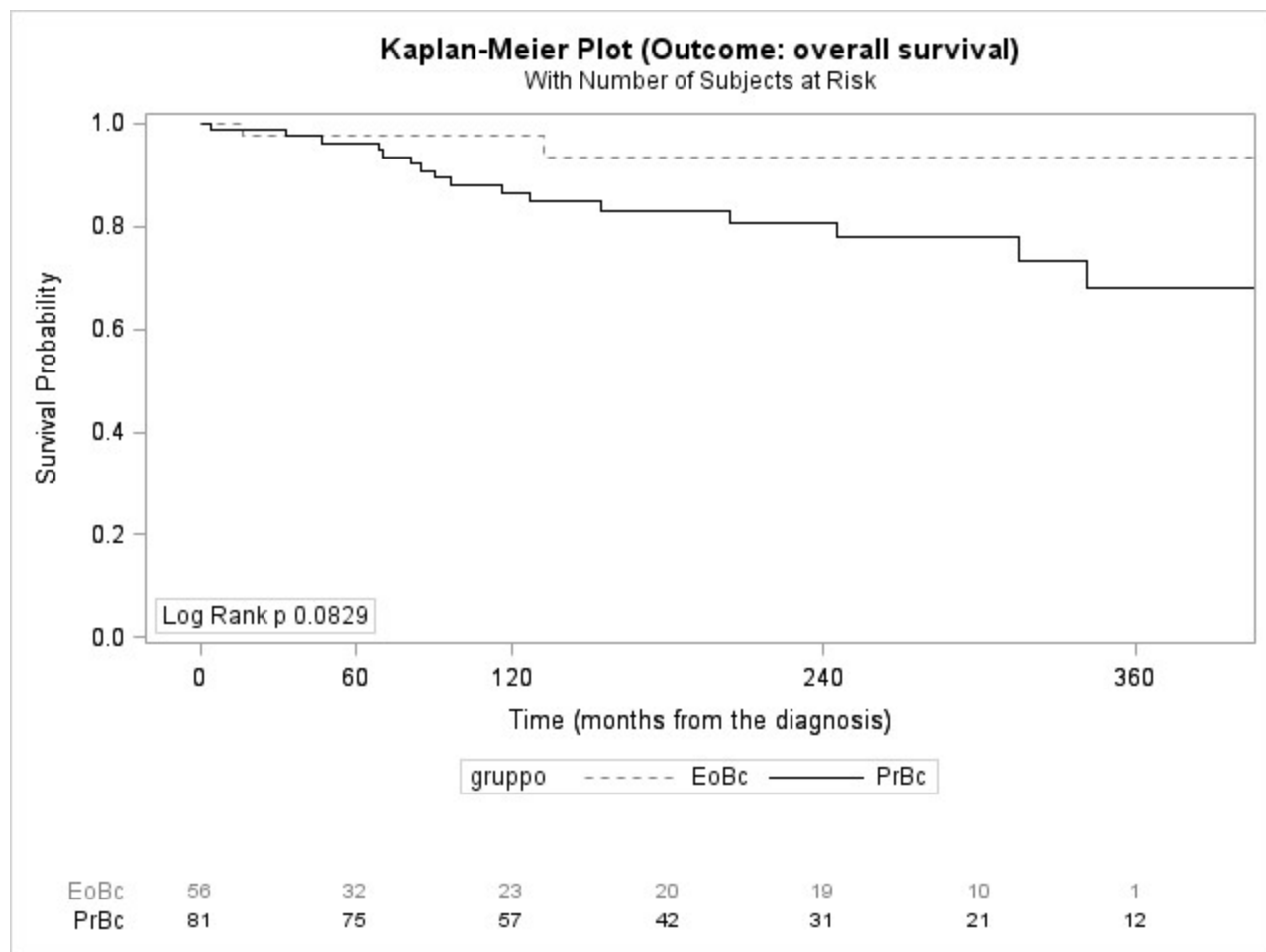

Supplement: Supplementary file 1 [file cells-11-02286-s001.zip › Supplementary Figure S2a_1.pdf]

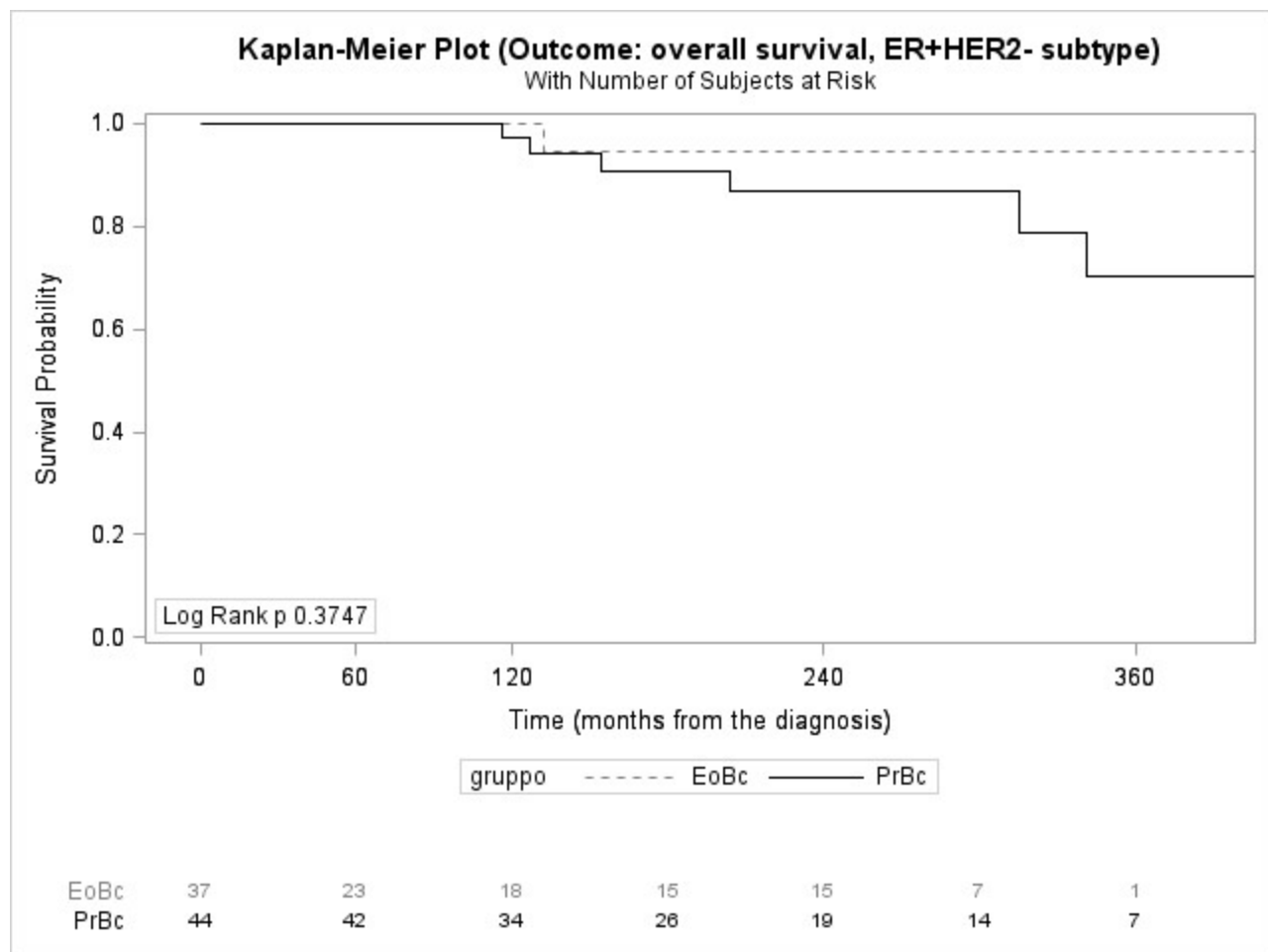

Supplement: Supplementary file 1 [file cells-11-02286-s001.zip › Supplementary Figure S2a_2.pdf]

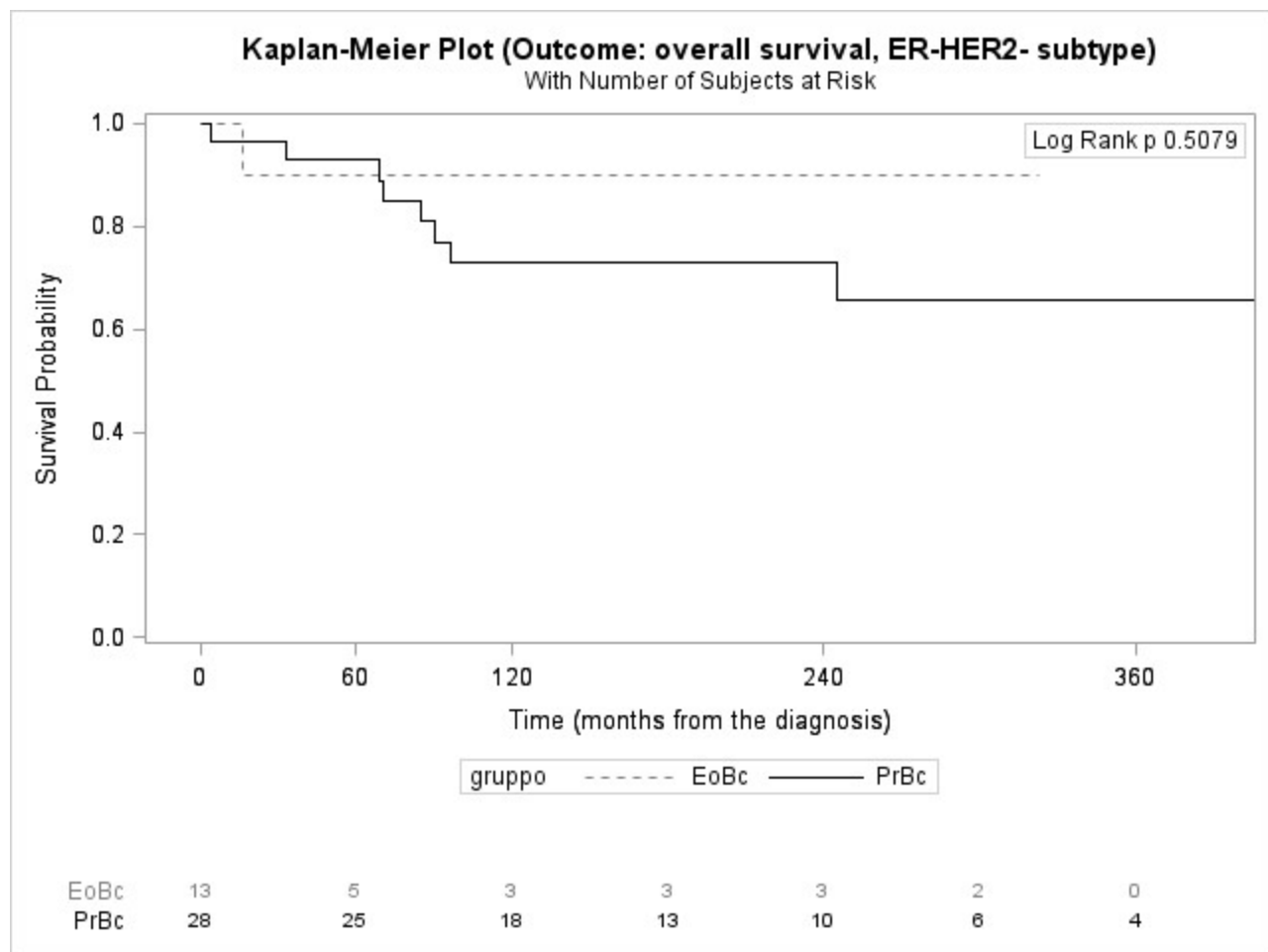

Supplement: Supplementary file 1 [file cells-11-02286-s001.zip › Supplementary FIgure S2a_3.pdf]

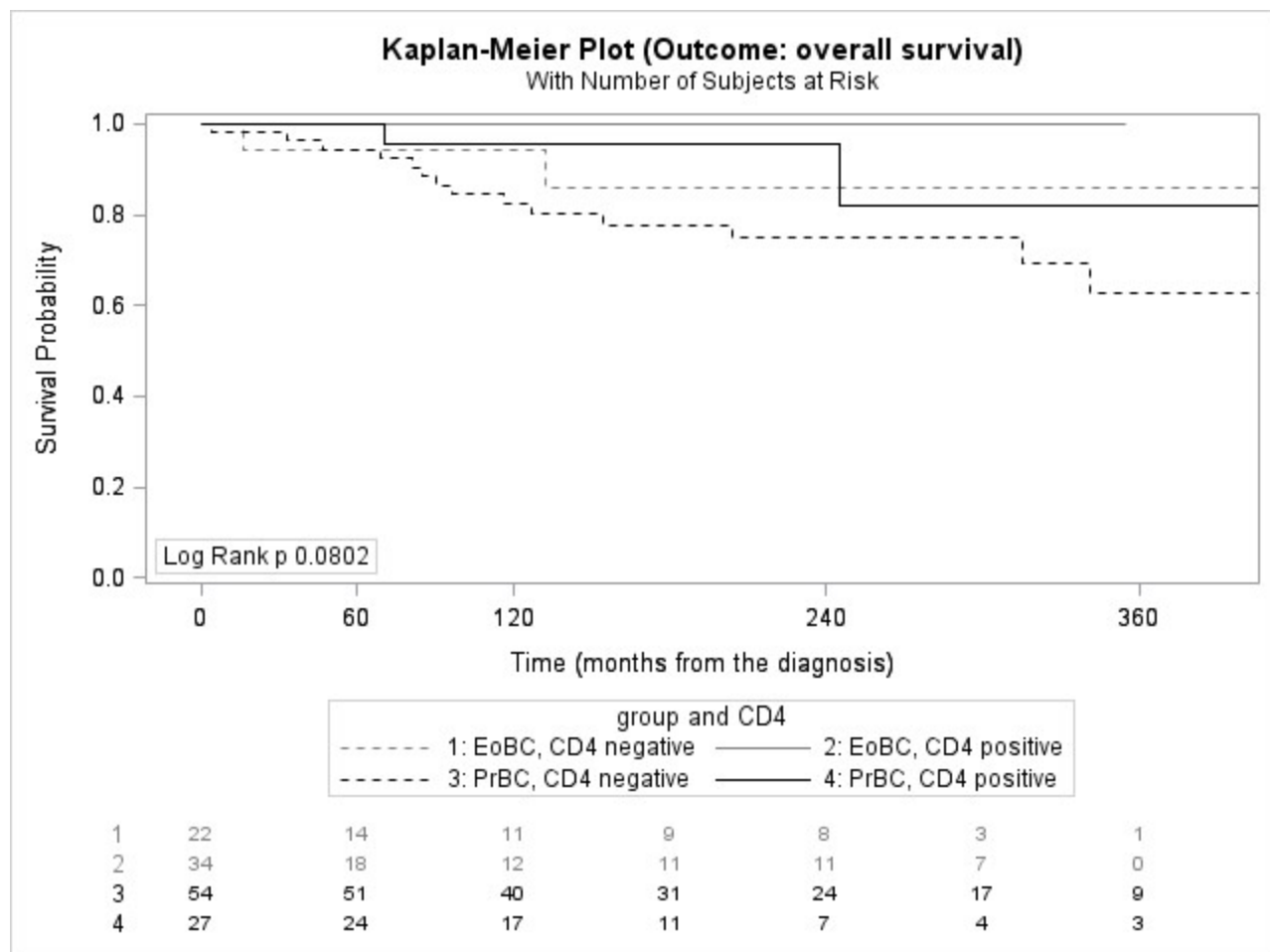

Supplement: Supplementary file 1 [file cells-11-02286-s001.zip › Supplementary Figure S2a_4.pdf]

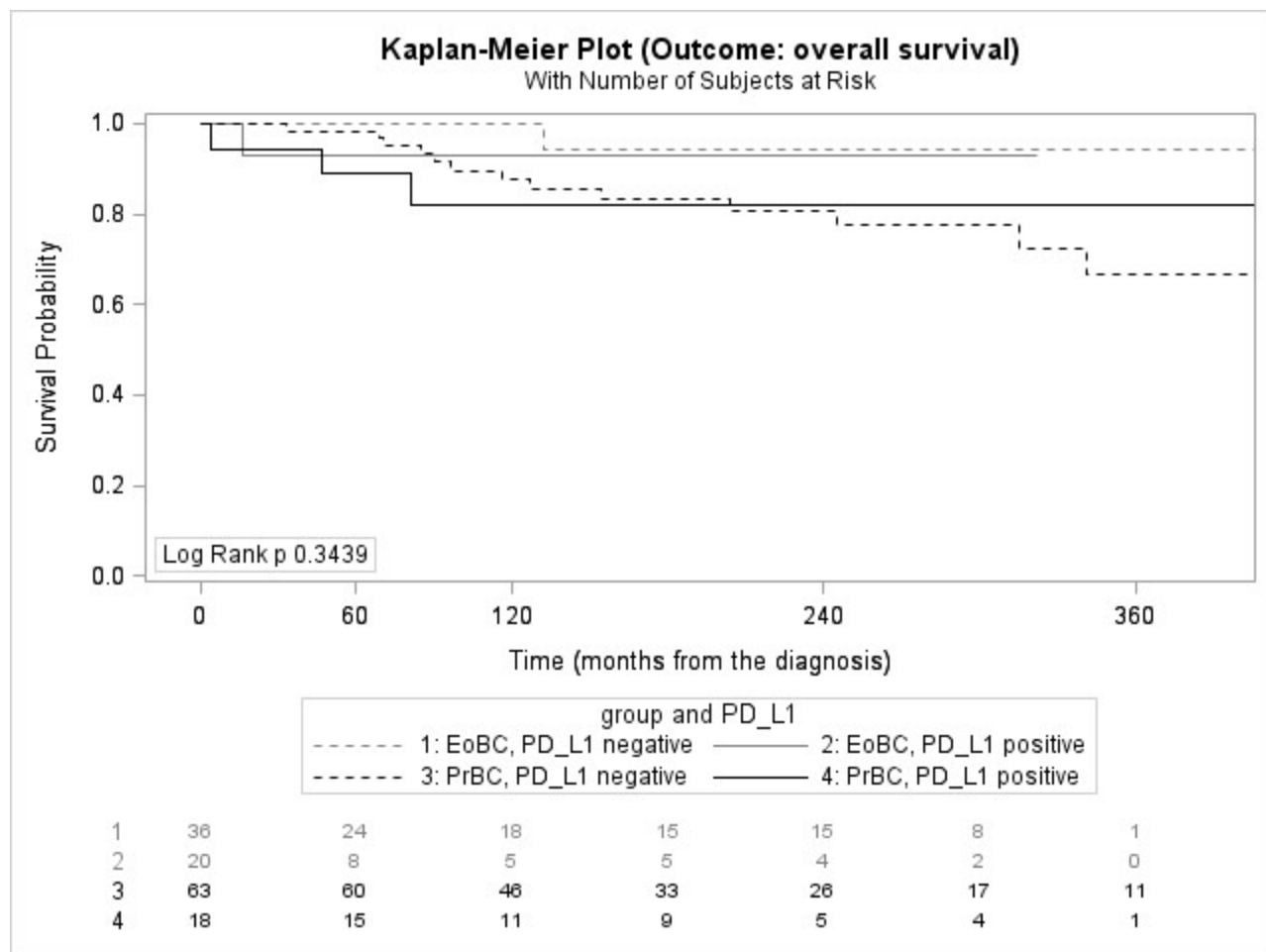

Supplement: Supplementary file 1 [file cells-11-02286-s001.zip › Supplementary Figure S2a_5.pdf]

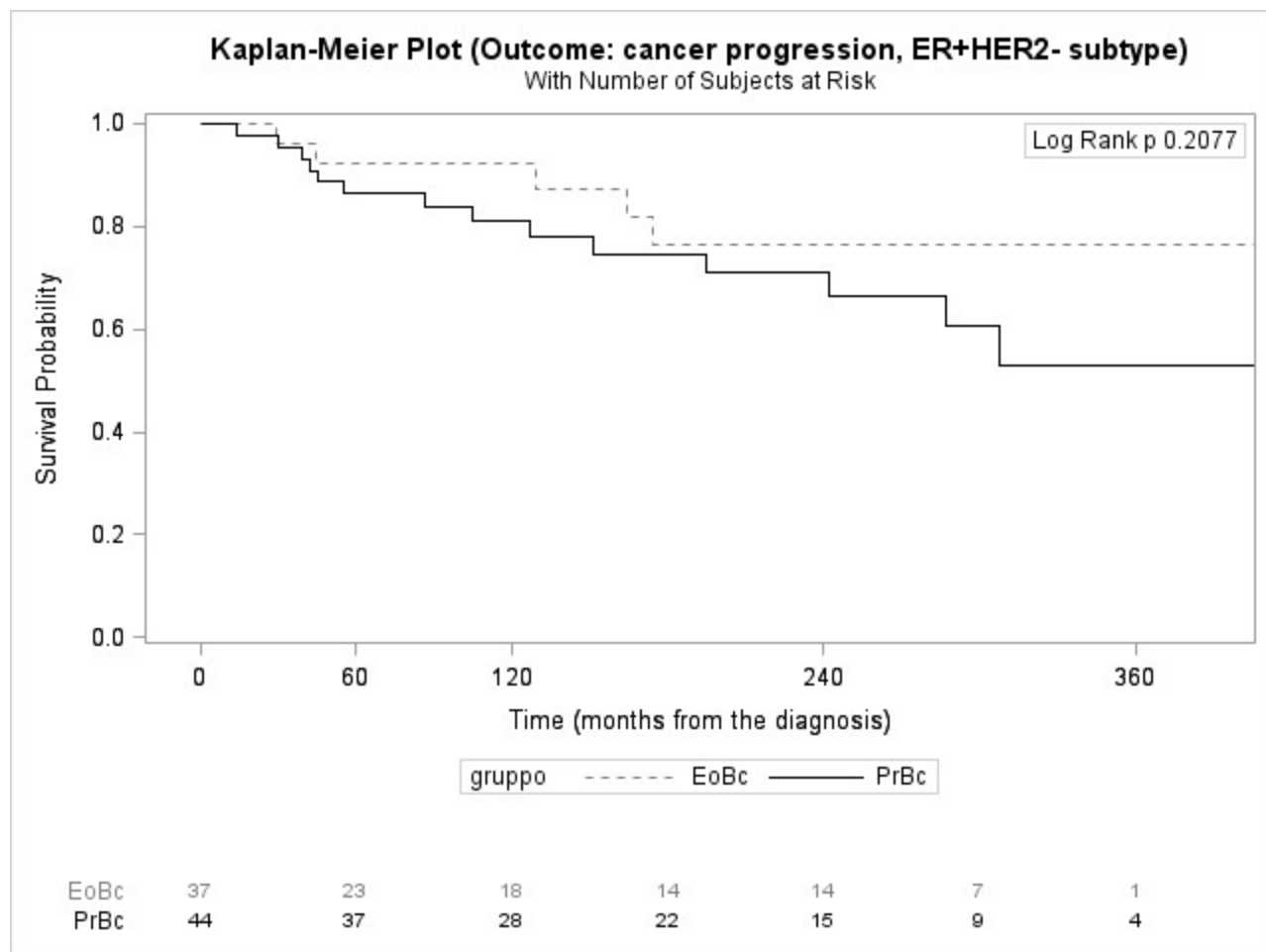

Supplement: Supplementary file 1 [file cells-11-02286-s001.zip › Supplementary Figure S2b_1.pdf]

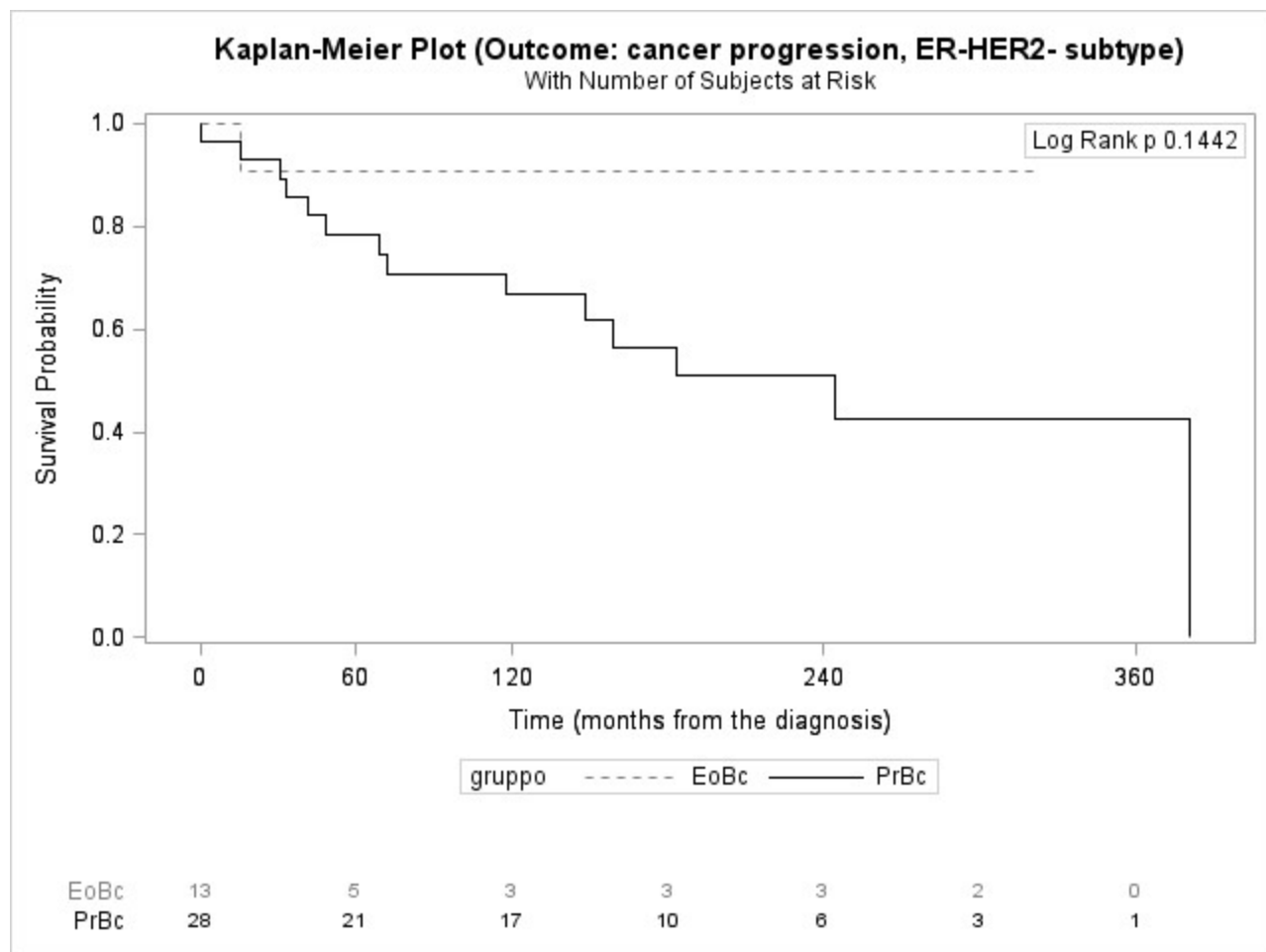

Supplement: Supplementary file 1 [file cells-11-02286-s001.zip › Supplementary Figure S2b_2.pdf]

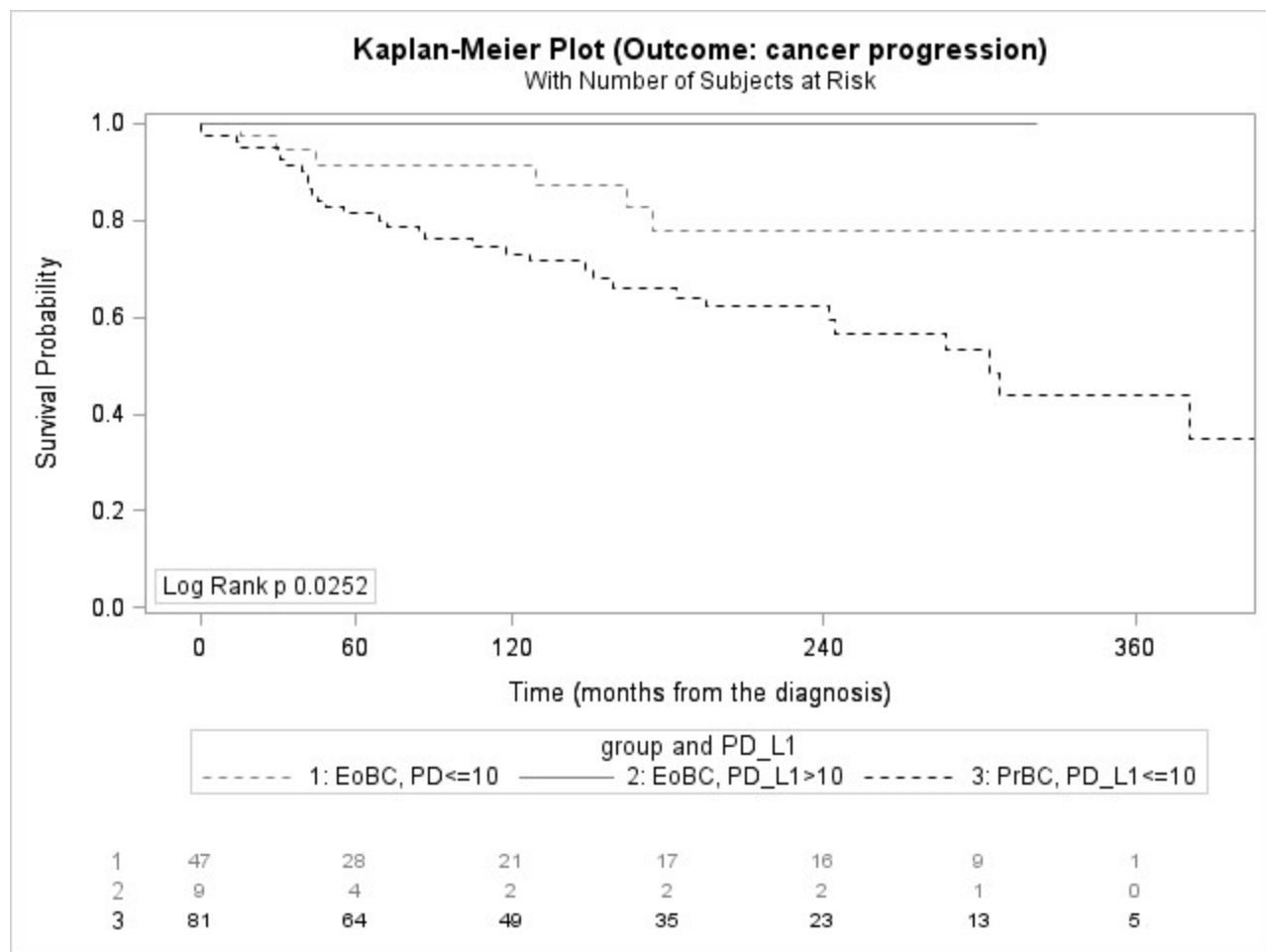

Supplement: Supplementary file 1 [file cells-11-02286-s001.zip › Supplementary Figure S2b_3.pdf]
